# Features of sRNA biogenesis in rice revealed by genetic dissection of sRNA expression level

**DOI:** 10.1016/j.csbj.2020.10.012

**Published:** 2020-10-23

**Authors:** Wen Yao, Yang Li, Weibo Xie, Lei Wang

**Affiliations:** a National Key Laboratory of Wheat and Maize Crop Science, College of Life Sciences, Henan Agricultural University, Zhengzhou 450002, China; b National Key Laboratory of Crop Genetic Improvement, National Center of Plant Gene Research (Wuhan), Huazhong Agricultural University, Wuhan 430070, China

**Keywords:** Small RNA, sRNA, sRNA biogenesis, QTL mapping, sRNA expression level, Rice

## Abstract

We previously conducted a QTL analysis of small RNA (sRNA) abundance in flag leaves of an immortalized rice F_2_ (IMF2) population by aligning sRNA reads to the reference genome to quantify the expression levels of sRNAs. However, this approach missed about half of the sRNAs as only 50% of all sRNA reads could be uniquely aligned to the reference genome. Here, we quantified the expression levels of sRNAs and sRNA clusters without the use of a reference genome. QTL analysis of the expression levels of sRNAs and sRNA clusters confirmed the feasibility of this approach. sRNAs and sRNA clusters with identified QTLs were then aligned to the high-quality parental genomes of the IMF2 population to resolve the identified QTLs into *local* vs. *distant* regulation mode. We were able to detect new QTL hotspots by considering sRNAs aligned to multiple positions of the parental genomes and sRNAs unaligned to the parental genomes. We found that several *local*-QTL hotspots were caused by sequence variations in long inverted repeats, which probably function as precursors of sRNAs, between the two parental genomes. The expression levels of these sRNAs were significantly associated with the presence/absence of the long inverted repeats in the IMF2 population. Moreover, we found that the variations in whole-genome sRNA species composition among different IMF2s were attributed to sRNA biogenesis genes including *OsDCL2b* and *OsRDR2.* Our results highlight that genetic dissection of sRNA expression is a promising approach to disclose new components functioning in sRNA biogenesis and new mechanisms of sRNA biogenesis.

## Graphical abstract

**Figure:**
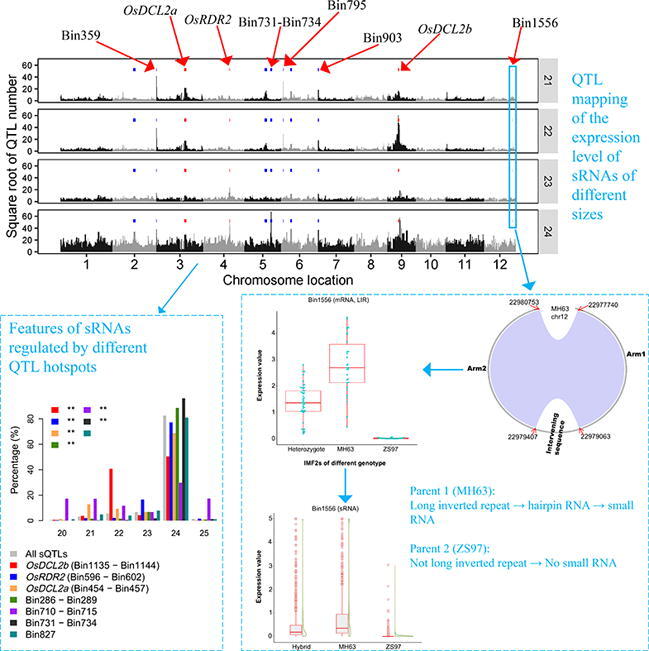

## 1 Introduction

Small RNAs (sRNAs) are non-coding RNAs with typical sizes of 18–30 nucleotides involved in diverse biological processes [1], [2]. sRNAs are categorized into two major classes, *hpRNA* (hairpin RNA) whose precursor is single-stranded hairpin RNA and siRNA (small interfering RNA) whose precursor is double-stranded RNA [1], [3]. microRNA (miRNA) is the major type of *hpRNA* that has been well studied in many plants and animals. In rice, OsmiR530 was reported to regulate grain yield through down-regulating of a target gene *OsPL3* [4]. Knockdown of another miRNA miR166 in rice enhanced drought resistance by reducing transpiration through regulation of stem vasculature and hydraulic conductivity [5]. Tuning the accumulation of miR528 in rice can modulate flowering time and the defense against *Rice stripe virus* by regulating different target genes in different pathways [6], [7]. siRNA could be further classified according to their evolutionary origin and further biogenesis steps. A siRNA derived from transposon elements was reported to be involved in disease resistance in rice [8]. Triggered by miRNA, phased siRNA (phasiRNA) is a type of siRNA broadly present in angiosperms and was reported to be involved in rice spikelet development [9], [10].

Dicer-like proteins (DCLs), RNA-dependent RNA polymerases (RDRs) and Argonautes (AGOs) are the key proteins functioning in the biogenesis of sRNAs [1], [11]. In addition to its role in the processing of miRNAs, *DCL1* was reported to be involved in the production of sRNAs from endogenous inverted repeats in *Arabidopsis* [12]. In rice, *OsDCL3a* was found to be responsible for the processing of 24-nt siRNAs from miniature inverted-repeat transposable elements (MITEs) [13]. Although the key components of sRNA biogenesis have been well studied, new species of sRNAs and new genes involved in sRNA biogenesis were frequently reported in recent years. For instance, sidRNA (siRNAs independent of DCLs) is a new class of siRNAs identified in *Arabidopsis*, which was reported to be involved in DNA methylation [14]. *CUE1*, which encodes the plastid inner envelope phosphoenolpyruvate, is a new gene that was found to regulate miRNA biogenesis in *Arabidopsis* by affecting the accumulation of primary and mature miRNAs [15].

Although many studies have been conducted to dissect the biological functions of sRNAs in various organisms, the genetic and molecular mechanisms underlying the quantitative variations among group of individuals with different genotypes were merely investigated [16]. In a previous study, we conducted quantitative trait locus (QTL) analysis of sRNA expression variations using all species of sRNAs of an experimental population in rice [16]. sRNA reads were aligned to the reference genome to define sRNA expression traits (s-traits) and sRNA cluster expression traits (sc-traits), which were used to identify QTLs regulating their expressions [16]. This approach could be designated as the “align-then-quantify” approach. Only sRNAs uniquely mapped to the reference genome were used in the analysis of the previous study.

In this study, we proposed a “quantify-then-align” approach. We defined s-traits by directly normalizing the number of sRNA reads and identified sc-traits by assembling sRNA reads, and used them to perform QTL mapping. sRNAs with detected QTLs were then aligned to the high-quality parental genomes of the experimental population. New QTL hotspots were identified and the genetic mechanisms underlying several QTL hotspots were investigated with the help of the high-quality genomes of the two parents. Features of sRNAs regulated by serval QTL hotspots were investigated, which shed light on the mechanisms of sRNA biogenesis in rice flag leaves.

## 2 Results

### 2.1 QTL analysis of s-traits identified by directly normalizing the read count of sRNAs

We previously reported the sRNA sequencing data of 98 IMF2s [16]. For each IMF2, the reads of all species of sRNAs were first aligned to the SNP-replaced reference genomes, in which the Nipponbare reference genome was used as the backbone and the SNP sites were replaced by the sequences of Zhenshan 97 or Minghui 63, which are the parents of the IMF2 population, respectively [17]. By so doing, a total of 53,613,739 unique sRNA sequences were identified, which included only<50% of the sRNAs from all sequencing libraries [Supplementary file 1 in Dryad [18]]. Thus, more than half of the sRNAs were left out from further analyses.

To recover as much as possible data in the analysis, we analyzed all the 136,080,320 unique sRNAs obtained by integrating data of all libraries, and found that about 88.66% of these sRNAs were present in no more than 5 IMF2s while only 0.12% were present in all 98 IMF2s (Fig. S1A). Approximately 43.99% of the sRNAs were 24 nt (Fig. S1B). The distribution of sRNAs in different genomic regions were surveyed in our previous study [16]. Here, we investigated the nucleotide composition at each base position of all sRNAs. Taking all base positions together, guanosine (G) is the most common while cytidine (C) is the rarest nucleotide for sRNAs of 21, 22 and 23 nt (Fig. S2A-C). For 24-nt sRNA, adenosine (A) is the most common while C is the rarest nucleotide incorporating all base positions (Fig. S2D). The nucleotide composition for sRNAs of diverse sizes varied across different base positions. Starting from the first base at the 5′ end, the percentage of A decreased sharply to the second base, and went down slightly till the third base at the 3′ end, and then augmented steeply towards the first two bases at the 3′ end (Fig. S2E-H). On the contrary, the proportion of G increased from the first base to the second base at the 5′ end, and went up slightly till the third base from the 3′ end, and then fell off steeply towards the first two bases at the 3′ end (Fig. S2 E-H). These two rules also held true for sRNAs of 21, 22, 23 and 24 nt.

The abundance of each sRNA in a library was normalized to number of reads per millions (RPM) to quantify the sRNA expression levels in each library (Materials and methods). sRNAs present in more than 48 IMF2s were considered as sRNA expression traits (s-traits). In total, 1,805,909 s-traits were obtained, reaching more than ten times of the number of s-traits in our previous study (Supplementary file 1). Recently, we reported the high-quality genome sequences of Zhenshan 97 and Minghui 63 [17]. The distribution of s-traits based on the genome sequence of Minghui 63 was similar to the results of our previous study (Fig. S3) [16]. The nucleotide composition at each base position of s-traits resembled that of the sRNAs (Fig. S2, S4).

**Fig. 1:**
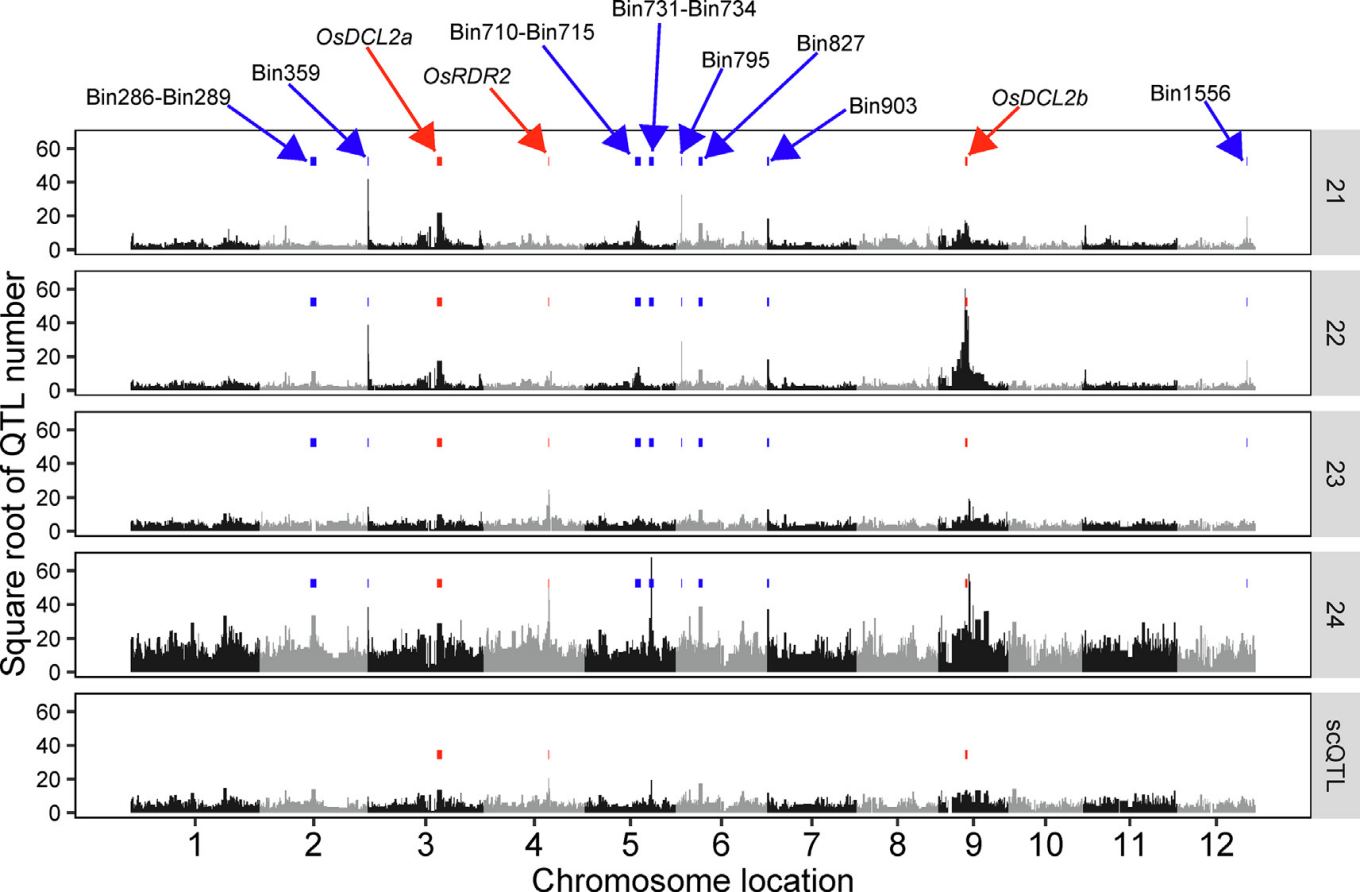
The distribution of sQTLs and scQTLs across the 1,567 bins. The top four panels are QTLs for sRNAs of 21, 22, 23 and 24 nt while the panel at the bottom shows the QTLs for sc-traits. The 1,567 bins are represented as black bars and are arranged from left to right based on their genomic positions. The width of each bar represents the size of the bin. The chromosome identifiers are labeled on the X-axis. Adjacent chromosomes are represented by different colors. Bins harboring sRNA biogenesis genes are indicated with red rectangles above the bins. Clusters of QTL regulating the expression of large numbers of sRNAs are indicated with blue rectangles above the bins. The bin IDs and the sRNA biogenesis genes for representative bins are indicated in the uppermost panel. (For interpretation of the references to color in this figure legend, the reader is referred to the web version of this article.)

We then conducted QTL analysis for these s-traits with a new genetic map constructed based on the genome of Minghui 63 using composite interval mapping (CIM) implemented in the R/qtl package with default parameters [19], [20], [21] (Supplementary file 2). QTLs with LOD score ≥ 5 was recovered, resulting in 517,495 QTLs for 495,232 s-traits, which were designated as sQTLs (QTLs regulating the expression of sRNAs) (Fig. 1, Supplementary file 3). Several sQTLs regulating the expression of plentiful sRNAs were located in or neighboring the bins harboring sRNA biogenesis genes including *OsRDR2*, *OsDCL2a* and *OsDCL2b,* which was consistent with the previous results (Fig. 1) [16]. Another 8 clusters of sQTLs, Bin286-Bin289, Bin359, Bin710-Bin715, Bin731-Bin734, Bin795, Bin827, Bin903 and Bin1556, were identified to explain the expression variations of large amounts of sRNAs (Fig. 1). Among these sQTLs, Bin795 and Bin1556 were newly identified in this study.

To compare the new approach with the approach adopted in our previous study, we further conducted QTL analysis of these s-traits based on the same genetic map used in our previous study and identified 517,945 QTLs for 496,423 s-traits. In all, 156 bins were identified to encompass 166,771 (32.2%) sQTLs; sQTLs residing in each of the bins regulated the expression of more than 600 s-traits. It was found that more than 73.7% of the 156 bins were in the list of the 200 bins that were identified to regulate the expression of the highest number of s-traits in our previous study [16].

### 2.2 QTL analysis of sc-traits obtained based on assembly of sRNA reads

In our previous study, we defined 80,362 sRNA clusters with the help of the reference genome and identified scQTLs (QTLs regulating the expression of sRNA clusters) regulating their expressions [16]. Here, we performed assembly of sRNA reads to obtain the DNA sequence from which a cluster of sRNAs was transcribed (Materials and methods). A total of 394,965 sRNA clusters were obtained (Supplementary file 4). The average length of all the sRNA clusters was 84 bp (ranging from 60 bp to 3487 bp) (Fig. S5). More than 89.90% and 89.52% of all sRNA clusters can be aligned to the genomes of Minghui 63 and Zhenshan 97, respectively, with ≥ 70% query coverage and ≥ 80% identity (BLASTN) (Fig. S6) [22]. Taking the alignments to both genomes together, more than 93.98% of all sRNA clusters can be aligned with ≥ 70% query coverage and ≥ 80% identity.

To obtain the read count of each sRNA cluster for all 98 IMF2s, we mapped the clean sRNA sequencing reads of each IMF2 to all the sRNA clusters using Bowtie (Materials and methods) [23]. The read count table was then normalized by DESeq [24] to define the expression level of each sRNA cluster. sRNA clusters with normalized expression value ≥ 6 in more than 25 of all the 98 IMF2s were regarded as sRNA cluster expression traits (sc-traits). As a result, 131,249 sc-traits were obtained, which was more than twice of the number of sc-traits identified in our previous study. QTL analysis was performed for all the sc-traits with the genetic map constructed based on the genome of Minghui 63 and 55,061 scQTLs (LOD score ≥ 5) were recovered for 51,606 sc-traits (Fig. 1, Supplementary file 5). Similarly, several QTLs regulating the expressions of large numbers of sc-traits were located in or neighboring bins harboring sRNA biogenesis genes including *OsDCL2a*, *OsRDR2* and *OsDCL2b* (Fig. 1).

We further performed QTL analysis for these sc-traits based on the same genetic map used in our previous study [16]. A total of 55,365 scQTLs (LOD score ≥ 5) were recovered for 51,822 sc-traits. About 15.5% of these scQTLs were mapped to 60 bins, and QTLs in each of these bins explained the variations in expression of more than 100 sc-traits. Moreover, 46 of these 60 bins were in the list of the 80 bins representing the highest number of scQTLs detected in our previous study.

### 2.3 Resolving sQTLs and scQTLs as *local*-QTLs and *distant*-QTLs by aligning the sequences of s-traits and sc-traits to the reference genome

We then classified sQTLs and scQTLs as *local*-QTLs and *distant*-QTLs based on the alignment of the sequences of s-traits and sc-traits with detected QTLs to the SNP-replaced reference genomes of the parents using Bowtie and BLASTN respectively (Materials and methods) [22], [23]. A *local*-QTL represents local functional polymorphism(s) affecting the expression of the target gene, while a *distant*-QTL indicates the expression of the target gene is controlled by regulatory element(s) distant from the target gene [16]. A total of 171,090 (53.7%) *local*-sQTLs and 147,353 (46.3%) *distant*-sQTLs were resolved for 306,371 s-traits, while 12,851 (55.7%) *local*-scQTLs and 10,227 (44.3%) *distant*-scQTLs were obtained for 22,070 sc-traits (Fig. S7, S8, S9, S10, Supplementary file 6, Supplementary file 7). We also identified hotspots of *local*-QTLs and *distant*-QTLs using the same definitions proposed in our previous study (Fig. S11, Supplementary file 8, Supplementary file 9). sRNA biogenesis genes including *OsDCL2a*, *OsRDR2* and *OsDCL2b* were observed in consecutive *distant*-sQTLs hotspots, in accordance with the results of our previous study (Fig. S11).

We next aligned all the 1,805,909 s-traits to the high-quality genomes of Zhenshan 97 and Minghui 63. A total of 88.59% and 89.03% s-traits can be aligned to one or multiple positions of the Minghui 63 and Zhenshan 97 genome without mismatch, respectively. Only 1,178,301 (65.24%) s-traits can be uniquely aligned without mismatch to either of the parental genomes. Taking the alignments to both genomes together, 96.21% (1,737,482) of all s-traits can be aligned without mismatch to the parental genomes (Fig. S12A). For the rest 68,427 s-traits, 52,575 (76.83%) can be aligned with one mismatch to one or multiple positions of the parental genomes (Fig. S12B). We further compared the genomic alignments of s-traits and the genomic positions of sQTLs regulating the s-traits. We found that more than 32% of the sQTLs were ≤ 1 Mb away from at least one alignment of the corresponding s-traits (Fig. S12C, D).

### 2.4 Features of sRNAs regulated by different *distant*-sQTL hotspots

**Fig. 2:**
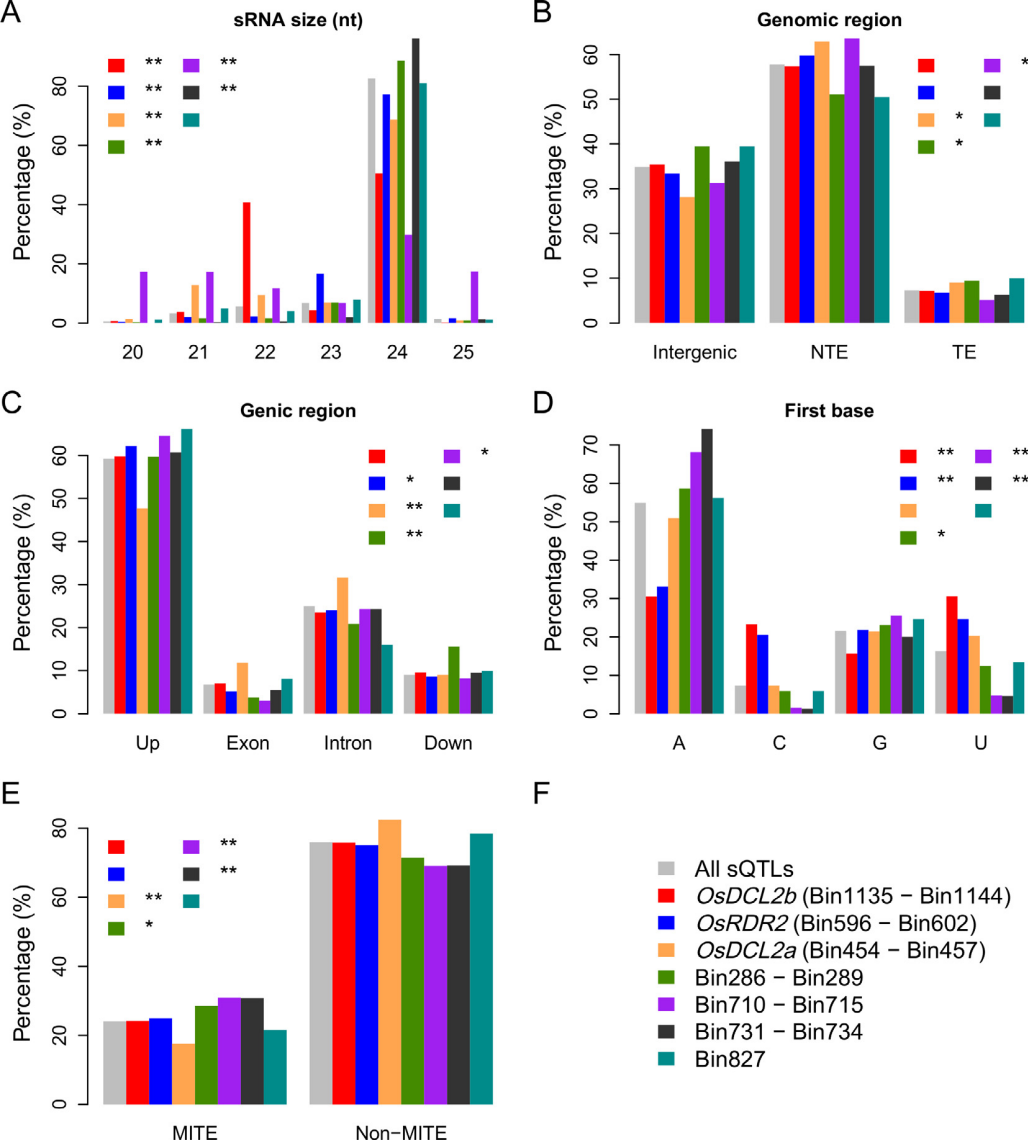
Diverse features of uniquely mapped sRNAs regulated by different sQTL hotspots. sRNAs regulated by each sQTL hotspot are categorized into different groups based on varying features of sRNAs, including the size of sRNAs (A), the genomic distribution of sRNAs (B), the genic distribution of sRNAs (C), the nucleotide preference at the first base position at the 5′ end (D), the origination of sRNAs from MITEs (E). Different sQTL hotspots are represented by different colors (F). All sRNAs with detected QTL are also grouped based on diverse feature of sRNAs and are labeled with grey color. The species of sRNAs in different groups are compared with that of all sRNAs with QTL using chi-squared test. *, *p*-value < 1e-5. **, *p*-value < 1e-10.

In our previous study, we found that 79.7% of the sRNAs regulated by the sQTL hotspot harboring *OsRDR2* were 24 nt while 51.0% of the sRNAs attributed to the sQTL hotspot harboring *OsDCL2b* were 22 nt, in consistent with the results of functional studies of *RDR2* and *DCL2* in *Arabidopsis* [16], [25]. Here, we surveyed the features of sRNAs regulated by seven different sQTL hotspots, including Bin286-Bin289, Bin454-Bin457 (harboring *OsDCL2a*), Bin596-Bin602 (harboring *OsRDR2*), Bin710-Bin715, Bin731-Bin734, Bin827 and Bin1135-Bin1144 (harboring *OsDCL2b*). Only sRNAs that were uniquely aligned to the Minghui 63 reference genome and the corresponding sQTLs that could be resolved into *local*-sQTL or *distant*-sQTL were included in the following analyses. Apart from Bin827, the proportion of sRNAs of different sizes regulated by the other six sQTL hotspots were significantly different from that of all sRNAs with detected QTLs (Fig. 2A). Compared with all sRNAs with detected QTLs, higher percentage of sRNAs regulated by Bin454-Bin457 were from non-transposon genes (Fig. 2B), especially the exon and intron of non-transposon genes (Fig. 2C). For five sQTL hotspots, the proportion of sRNAs beginning with different nucleotide at 5′ end was significantly different from that of all sRNAs with detected QTLs (Fig. 2D). We found that the 5′ first base of higher proportions of 21-nt and 22-nt sRNAs were U, compared with all sRNAs and larger portion of sRNAs regulated by Bin710-Bin715 were of 21 and 22 nt (Fig. 2A). However, the 5′ first base of 68.1% of sRNAs regulated by Bin710-Bin715 were A, a proportion higher than that of all sRNAs with QTLs (Fig. 2D). Moreover, higher proportion of sRNAs regulated by Bin710-Bin715 and Bin731-Bin734 were from MITEs, compared with all sRNAs with QTLs (Fig. 2E). In contrast, lower proportion of sRNAs regulated by Bin454-Bin457 were from MITEs. Excluding *local*-sQTLs, the features of all the sRNAs regulated by the seven sQTL hotspots remained unchanged in general (Fig. S13). We further explored the size and the nucleotide at the first base position at 5′ end for all the sRNAs, including ones that couldn’t be uniquely aligned to the Minghui 63 reference genome, regulated by the sQTL hotspots. The results were generally similar to that of all sRNAs uniquely aligned to the reference genome (Fig. S14).

### 2.5 Several *local*-sQTL hotspots were caused by variations in long inverted repeats between the genomes of Minghui 63 and Zhenshan 97

**Fig. 3:**
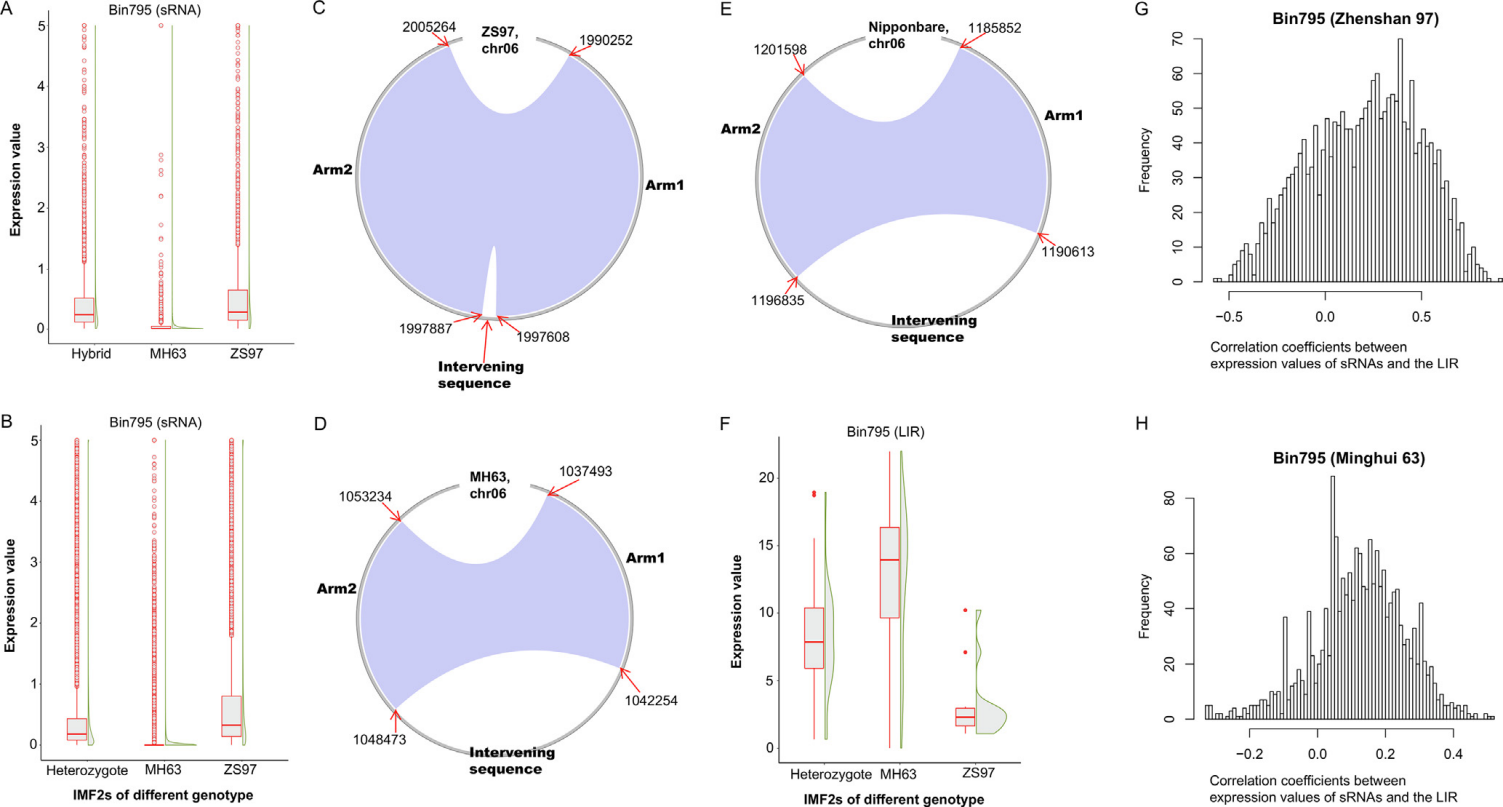
sRNAs regulated by Bin795 and the long inverted repeat functioning as the potential precursors of these sRNAs. (A) Expression values of sRNAs regulated by Bin795 in Zhenshan 97 (ZS97), Minghui 63 (MH63) and the hybrid. (B) Expression values of sRNAs regulated by Bin795 in IMF2s of different genotypes. ZS97, the Zhenshan 97 genotype. MH63, the Minghui 63 genotype. Heterozygote, heterozygote genotype. (C) Structure of the long inverted repeat (LIR) (chr06:1989522–2005555) in the Zhenshan 97 genome created using shinyCircos [44]. The clockwise grey circle indicates the LIR. The two complementary regions are connected by the blue ribbon. (D) Structure of the LIR (chr06:1037057–1054781) in the Minghui 63 genome. (E) Structure of the LIR (chr06:1185416–1203145) in the Nipponbare genome. (F) Expression values of the LIR in IMF2s of different genotypes quantified by mRNA sequencing. (G) Correlation coefficients between expression values of sRNAs regulated by Bin795 and the LIR in IMF2s of Zhenshan 97 genotype. (H) Correlation coefficients between expression values of sRNAs regulated by Bin795 and the LIR in IMF2s of Minghui 63 genotype. (For interpretation of the references to color in this figure legend, the reader is referred to the web version of this article.)

Two new sQTL hotspots represented by Bin795 and Bin1556 were identified in this study (Fig. 1). The expressions of 2473 sRNAs were regulated by Bin795 (chr06: 1894025–2056990 in Minghui 63; chr06:1844230–2032924 in Zhenshan 97). A total of 43.1%, 34.0% and 20.4% of the 2473 sRNAs were of 21, 22 and 24 nucleotides. For more than 83.2% of the 2473 sRNAs, each could be aligned to two different positions within chr06:1989522–2005555 of the Zhenshan 97 genome. On the contrary, each one of a set of 793 sRNAs, among all 2473 sRNAs, could be aligned to two different positions within chr06:1037057–1054781 of the Minghui 63 genome, while each of another set of 1115 sRNAs could be aligned to a single position of this region. The expression values of 79.5% of the 2473 sRNAs were higher than 0.1 in the genome of Zhenshan 97 while the expression values of 81.8% sRNAs were lower than 0.05 in the genome of Minghui 63 (Fig. 3A). In addition, the expression levels of the majority of the 2473 sRNAs were close to zero in IMF2s of Minghui 63 genotype in this region (Fig. 3B). On the contrary, the expression levels of the majority of the 2473 sRNAs were higher than zero in IMF2s of Zhenshan 97 or heterozygote genotype in this region. We then compared the sequences of the two regions in the genomes of Minghui 63 and Zhenshan 97. The genomic region in Zhenshan 97 (chr06:1989522–2005555) is a long inverted repeat (LIR) composed of a sequence (chr06:1990252–1997608) and its reverse complement (chr06:1997887–2005264) separated by a short intervening sequence (chr06:1997609–1997886), which could probably form a *hpRNA* when transcribing (Fig. 3C). The genomic region in Minghui 63 genome is also a long inverted repeat composed of a sequence (chr06:1037493–1042254) and its reverse complement (chr06:1048473–1053234) separated by a very long intervening sequence (chr06:1042255–1048472) (Fig. 3D). The sequence of the corresponding region in the Nipponbare genome is the same to that of the Minghui 63 genome (Fig. 3E). We further quantified the expression levels of the LIR in the IMF2 population (Materials and methods). The expression values of the LIR were higher than zero in all 98 IMF2s (Fig. 3F). Contrary to the expressions of sRNAs, the expressions of the LIR in IMF2s of Minghui 63 genotype were higher than that in IMF2s of Zhenshan 97 genotype (Fig. 3F). The correlation coefficients between the expressions of sRNAs regulated by Bin795 and the LIR were calculated and more than 60% of the correlation coefficients were higher than 0 (Fig. 3G-H). These results implied that the genomic region in Minghui 63 genome probably cannot form an *hpRNA* due to the very long intervening sequence. The expression variations of these sRNAs among different IMF2s were probably attributed to the sequence divergence between the genomes of Zhenshan 97 and Minghui 63, which were detected by QTL analysis in this study. This sQTL hotspot would not be detected using solely uniquely aligned sRNA reads, and would not be illustrated using the Nipponbare reference genome. Nevertheless, we did observe the discordance between the expressions of the LIRs and the sRNAs regulated by Bin795. We found that the discordance was possibly caused by a genomic region on chromosome 1 highly similar to the LIR on chromosome 6 in the genome of Minghui 63, which hindered the accurate quantification of the LIR.

**Fig. 4:**
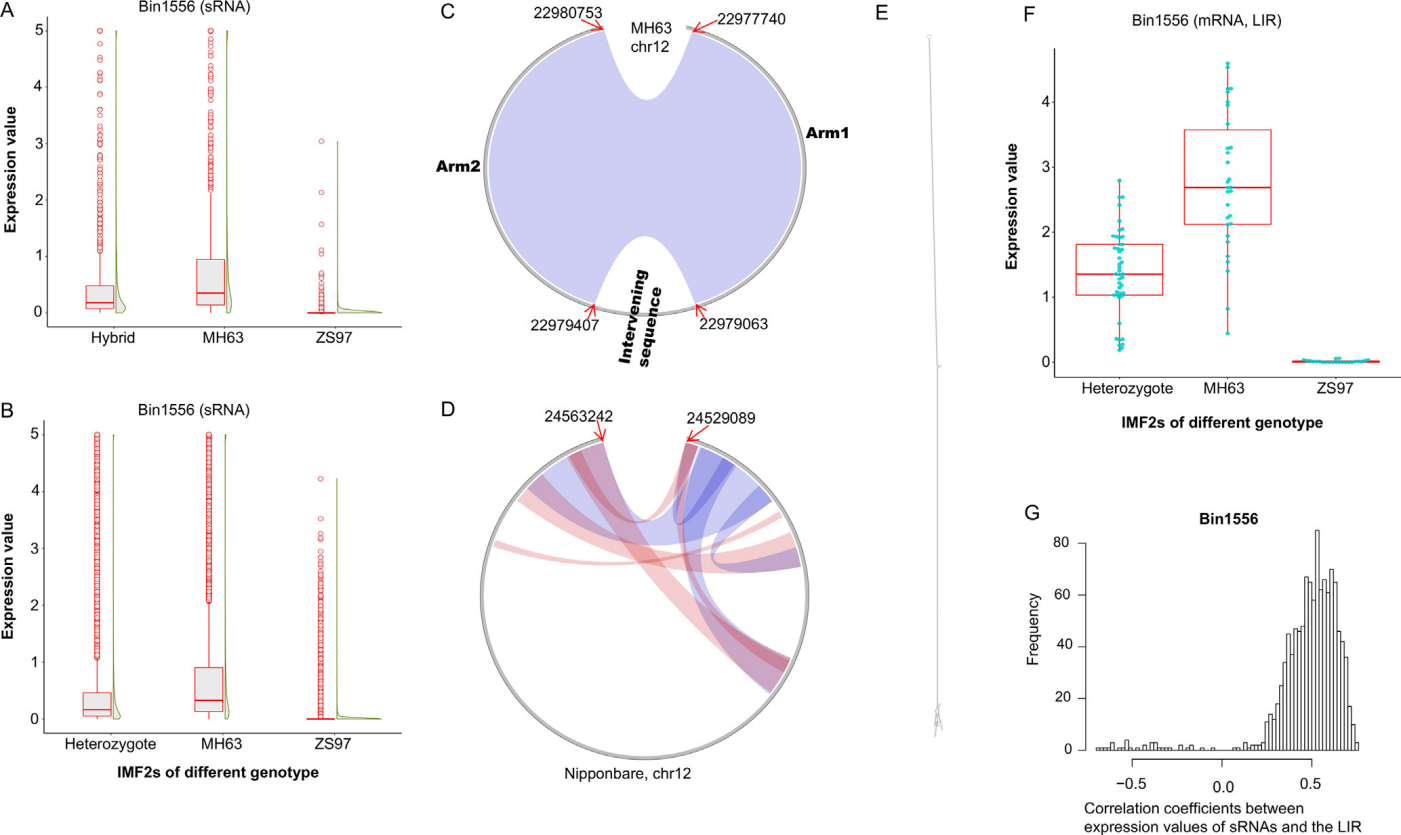
sRNAs regulated by Bin1556 and the long inverted repeat functioning as the potential precursors of these sRNAs. (A) Expression values of sRNAs regulated by Bin1556 in Zhenshan 97 (ZS97), Minghui 63 (MH63) and the hybrid. (B) Expression values of sRNAs regulated by Bin1556 in IMF2s of different genotypes. ZS97, the Zhenshan 97 genotype. MH63, the Minghui 63 genotype. Heterozygote, heterozygote genotype. (C) Structure of the long inverted repeat (LIR) (chr12:22977717–22980753) in the Minghui 63 genome. The clockwise grey circle indicates the LIR. The two complementary regions are connected by the blue ribbon. (D) Structure of the LIR (chr12:24529089–24563242) in the Nipponbare genome. (E) Predicted secondary structure of RNA encoded by chr12:22977717–22980753 of the Minghui 63 genome. (F) Expression values of the LIR in IMF2s of different genotypes quantified by mRNA sequencing. (G) Correlation coefficients between expression values of sRNAs regulated by Bin1556 and the LIR in 98 IMF2s. (For interpretation of the references to color in this figure legend, the reader is referred to the web version of this article.)

The expression of 1139 sRNAs, including 382 (33.5%), 314 (27.6%) and 369 (32.4%) sRNAs of 21, 22 and 24 nt, were regulated by another sQTL hotspot Bin1556 (chr12:22946937–23029125 in Minghui 63; chr12:23880702–23936203 in Zhenshan 97). For a set of 549 sRNAs out of all 1139 sRNAs, each one could be aligned to two different positions within chr12:22977717–22980753 of the Minghui 63 genome. Moreover, each one of another set of 339 sRNAs could be aligned to a single position of this region in the Minghui 63 genome. On the contrary, each one of a set of 482 sRNAs could be aligned to a single position within chr12:23895468–23896801 of the Zhenshan 97 genome. However, none of the 1139 sRNAs could be aligned to multiple positions of this region in the Zhenshan 97 genome. The expression values of the majority of all 1139 sRNAs were close to zero in Zhenshan 97 or IMF2s with Zhenshan 97 genotype but were higher than zero in Minghui 63 or IMF2s with Minghui 63 genotype (Fig. 4A-B). With the availability of the high-quality genomes of Minghui 63 and Zhenshan 97, we found that the genomic region in Minghui 63 is a LIR composed of a sequence (chr12:22977740–22979063) and its reverse complement (chr12:22979407–22980753) separated by a short intervening sequence (chr12:22979064–22979406), which could probably form an *hpRNA* (Fig. 4C). The genomic region in Zhenshan 97 is only a sequence highly similar to half of the corresponding genomic region in Minghui 63 without its reverse complement. The sequence in the Nipponbare genome in this region was quite different from that of Minghui 63 and Zhenshan 97 (Fig. 4D). The RNA sequence encoded by chr12:22977717–22980753 of the Minghui 63 genome were predicted as a long *hpRNA* as shown in Fig. 4E [26]. The expression values of the LIR were close to zero in IMF2s of Zhenshan 97 genotype but were significantly higher than zero in IMF2s of Minghui 63 or heterozygote genotype (Fig. 4F). The majority of the correlation coefficients between the expressions of sRNAs regulated by Bin1556 and the LIR were higher than 0 (Fig. 4G). These results implied that the expression variations of these sRNAs among different IMF2s were attributed to the sequence divergence between Zhenshan 97 and Minghui 63. This sQTL hotspot would not be detected using solely uniquely aligned sRNAs or using the Nipponbare reference genome. The mechanism underlying this sQTL hotspot would not be dissected without the available of the genome sequences of Minghui 63 and Zhenshan 97.

The two *local*-sQTL hotspots designated as Bin358 and Bin969 identified in the previous study were also recovered in this study, which were represented by Bin359 and Bin903 [16]. The majority of sRNAs regulated by Bin903 could be aligned to a genomic region on chromosome 7 (chr07:159842–163153 in Zhenshan 97; chr07:139399–142701 in Minghui 63). The expression value of sRNAs regulated by Bin903 were mostly higher than zero in Zhenshan 97, Minghui 63 and all IMF2s (Fig. S15A-B). This region in Zhenshan 97 and Minghui 63 were both long inverted repeats that could probably form *hpRNA*s (Fig. S15C-E). We speculate that the expression variations in sRNAs regulated by Bin903 are probably caused by the expression variations of the long inverted repeat, which could function as precursor of sRNAs, between the genomes of Zhenshan 97 and Minghui 63. We further found that the situation of Bin359 was similar to that of Bin903.

### 2.6 Contribution of sRNA biogenesis genes to the variation of whole-genome sRNAs species composition among different IMF2s

**Fig. 5:**
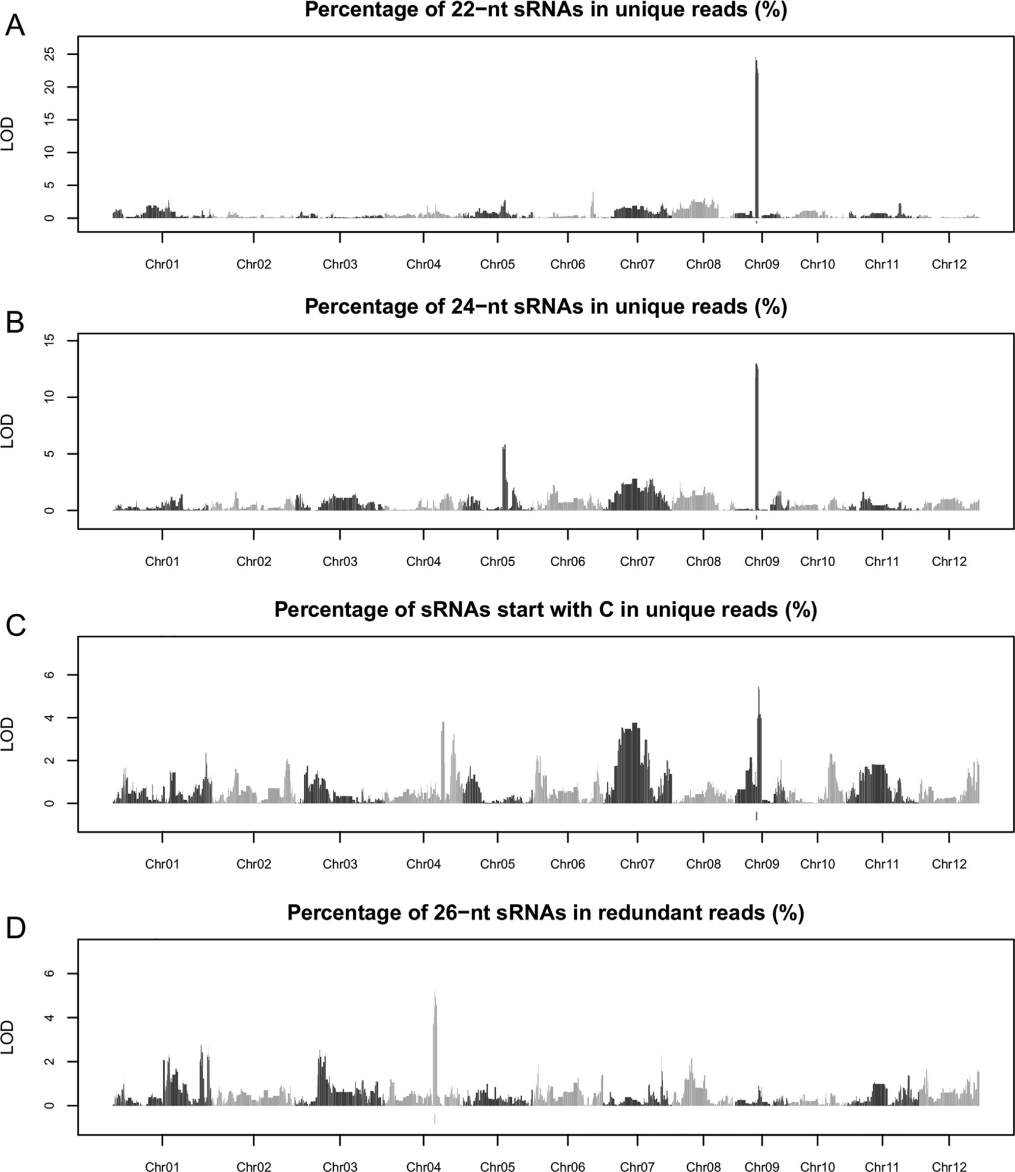
QTL analysis of variations in whole-genome sRNA species composition among different IMF2s. The 1,567 bins (QTL) are represented as vertical bars and arranged from left to right based on their genomic positions. The height of each bar indicates the LOD value of each bin while the width of each bar indicates the size of each bin. Adjacent chromosomes are denoted with different colors. The red rectangle in chromosome 9 indicates the bin harboring *OsDCL2b* while the red rectangle in chromosome 4 indicates the bin harboring *OsRDR2.*

We found that the whole-genome sRNAs species composition varied significantly among different IMF2s (Fig. S16). For instance, the percentage of 21-nt sRNAs in all sRNAs varied from 5.6% to 10.6% with an average of 7.8% among different IMF2s. We further detected extensive variations in the proportion of sRNA sequencing reads with specific features out of all sequencing reads, among different IMF2s (Fig. S17). We then conducted QTL analysis of these variations using CIM. A total of 20 QTLs were detected for 16 traits and the LOD value of 9 QTLs passed the threshold determined by 1,000 permutations (Supplementary file 10). *OsDCL2b* was frequently found in or neighboring the confidence interval of identified QTLs with the highest LOD values (Fig. 5, Supplementary file 10). In addition, *OsDCL2a* and *OsRDR2* were found in QTLs regulating the proportions of 21-nt and 26-nt sRNAs in the IMF2 population, respectively (Fig. 5, Supplementary file 10). These results suggested that sRNA biogenesis genes including *OsDCL2b*, *OsDCL2a* and *OsRDR2* contributed largely to the variation of whole-genome sRNAs species composition among different IMF2s in the flag leaves of rice.

## 3 Discussion

In this study, we proposed a “quantify-then-align” approach for QTL analysis of sRNA expression levels and demonstrated the feasibility of quantifying the expression levels of sRNAs and sRNA clusters, referred to as s-traits and sc-traits, without the use of a reference genome. With a molecular marker linkage map, QTL analysis can be performed for these s-traits and sc-traits exactly like any of the agronomic quantitative traits. Thus, for an organism without a reference genome, the strategy we proposed in this study can be applied to perform genetic analyses to identify QTLs regulating sRNA expression levels, although they could not be resolved into *local*- vs. *distant*- sQTLs and scQTLs, like in the case with an available reference genome. For an organism with a reference genome, the approach we explored here served as an alternative for conducting QTL analyses of sRNA expression levels, which also captured the genetic regulation of sRNAs not uniquely mapped to the reference genome as well as sRNAs unable to be aligned to the reference genome. This approach is much straightforward compared with traditional methods, as we can dissect the expression regulations of all species of sRNAs and identify QTL hotspots without choosing the appropriate strategy to align the sRNA reads, which was illustrated in Fig. 1. As for the mechanism underlying specific QTL hotspot of interest, we can then seek help form all aspects including investigations on the features and the genomic alignments of sRNAs regulated by the QTL hotspot, as well as additional genetic and molecular experiments.

Using the new approach, we were able to detect new QTL hotspots. With the high-quality sequences of the two parental genomes, we found that several *local*-sQTL hotspots including Bin795 and Bin1556 were attributed to the sequence variations in long inverted repeats, which probably functioned as precursors of sRNAs, between the parental genomes. This wouldn’t have been revealed using a single reference genome or by restricting the alignment of sRNAs to unique positions of the reference genome. The 2473 sRNAs, represented by tens of thousands of sRNA sequencing reads of the IMF2 population, regulated by Bin795 can be aligned without mismatch to 8076 positions across the 12 chromosomes of the Minghui 63 genome. Only 2701 of all 8076 alignments to chr06:1037057–1054781 of the Minghui 63 genome were determined as authentic alignments based on the results of QTL analysis and the alignments of these sRNAs to the Zhenshan 97 genome. If we used the “align-then-quantify” approach and assigned a random position for sRNA reads aligned to multiple positions, we would probably miss this QTL hotspot and the underlying mechanism. With the development of next-generation sequencing and the application of QTL mapping and genome-wide association studies, the approach we proposed in this study will be helpful for future researches on sRNA biogenesis. Using technologies of transcriptome-wide association studies, we can directly build the associations between sRNA expression variations and phenotypic variations to dissect the molecular functions of sRNAs, without using a reference genome [27].

After the discovery of DCLs, RDRs and AGOs as the core enzymes involved in sRNA biogenesis, hundreds of studies to uncover the functions of these enzymes in animals and plants were conducted [11]. Generally, individuals with knockout or knockdown of these enzymes were generated utilizing reverse genetic tools and the sRNA sequencing of these individuals were then compared with that of the wild types. In this study, we explored the genetic regulation of sRNA expression level using forward genetics approaches and investigated the connection between genomic regions regulating sRNAs expression levels and the diverse features of corresponding sRNAs. The length distribution of sRNAs regulated by sQTL hotspots harboring *OsRDR2* and *OsDCL2b* observed in our previous study and confirmed in this study, was in accordance with the results of functional studies on *RDR2* and *DCL2* in *Arabidopsis* [16], [25]. Compared with all sRNAs with QTL, higher portion of sRNA regulated by *OsDCL2b* started with U at the 5′ end. In *Arabidopsis*, the products of *DCL2* are loaded into *AGO1* which preferentially recruit sRNAs with a 5′ terminal U [28], [29], [30], [31]. In addition, we found that the variations in whole-genome sRNA species composition in the flag leaf of rice among different IMF2s was mostly determined by *OsDCL2b*, *OsDCL2a* and *OsRDR2*. Previously, we identified SNPs and indels in the genic and promoter regions of *OsDCL2a*, *OsDCL2b* and *OsRDR2* between the two parental genomes of the IMF2 population [16]. We also found that *OsDCL2a*, *OsDCL2b* were significantly differentially expressed between the two parental genomes of the IMF2 population. However, additional genetic and molecular experiments are required to disclose the functional variations responsible for the regulations of sRNA expression levels by these enzymes. These results are helpful for future dissection of the functions of these enzymes in rice as well as in other crops and shed light on the biogenesis mechanisms of sRNAs in rice.

## 4 Conclusions

Previously, we conducted QTL analyses of the expression levels of sRNAs in flag leaves of a rice IMF2 population. In this study, we proposed a new approach for analysis of sRNA sequencing data. The feasibility of this approach was verified by analysis of the sRNA sequencing data published in the previous study. Using the new approach, we were able to detect new QTL hotspots regulating the expression levels of sRNAs. Sequence variations in long inverted repeats between the parental genomes of the IMF2 population were found to be responsible for two new QTL hotspots. We further investigated the features of sRNAs regulated by different QTL hotspots. The results of this study provide new approaches for analysis of sRNA sequencing data and new insights into the biogenesis mechanisms of sRNAs in rice.

## 5 Materials and methods

### 5.1 Quantification of sRNA expression level

Clean sRNA reads obtained after a series of filtering in our previous study were used to quantify the expression level of sRNAs [16]. The expression level of a sRNA in a specific library was defined as the read count of this sRNA divided by the total number (in millions) of all sRNA reads in this library, which was designated as “reads per million” (RPM).

### 5.2 Definition of sRNA clusters based on assembly of sRNA reads

Assembly of sRNA reads was rarely reported especially for eukaryotes. Kreuze et al. [32] compared the assembly of sRNA reads in virus using SSAKE [33], VCAKE [34] and Velvet [35], and found that Velvet was faster and more accurate than SSAKE or VCAKE. We compared the assembly of our sRNA sequencing data using the three programs. We failed to run Velvet, as it required a very large amount of memory when processing our sRNA sequencing data. The results of SSAKE was generally better than that of VCAKE based on the N50 of assembled contigs and the alignments of assembled contigs to the rice reference genome. In addition, we tried to use other assemblers including Edena [36] and SHARCGS [37] which were reported to be suitable for the assembly of very short next generation sequencing reads. However, we failed to run Edena or SHARCGS with our data because they required all the sequencing reads to be the same length. As a result, we chose to perform assembly using SSAKE (parameters “-w 1 –m 16 –o 1 –z 50”) for sRNA sequencing reads of each IMF2 and the mixed reads of all 98 IMF2s [33]. All the assemblies of SSAKE were further clustered and assembled using GICL with default parameters [38], [39]. Contigs shorter than 60 bp were further removed.

### 5.3 Quantification of sRNA cluster expression level

The clean sRNA sequencing data of each IMF2 were aligned to the sequences of all sRNA clusters using Bowtie (parameters “-v 0 –m 1”) to obtain the read count for each sRNA cluster [23]. The R package DESeq [24] was used to normalize the read count of sRNA clusters to obtain the expression levels of sRNA clusters.

### 5.4 Construction of genetic map based on the genome of Minghui 63

The 98 IMF2s used in this study were obtained by paired crosses of 196 recombinant inbred lines (RILs) chosen from 210 RILs derived by single seed descent from a cross between Zhenshan 97 and Minghui 63 [40]. In previous studies, we conducted DNA sequencing of the 210 RILs and proposed a new method based on hidden Markov model to build an ultrahigh-density linkage map for the RIL population utilizing the Nipponbare reference genome [41], [42]. In this study, we built the genetic map for the 210 RILs using the method proposed in our previous study by aligning the DNA sequencing data to the Minghui 63 reference genome [17], [41]. A total of 262,749 high-quality SNPs were identified and were used to construct a genetic map consist of 1567 bins with each bin representing a genomic region composed of SNPs without recombination. The boundary between two adjacent bins indicates the genomic position where a recombination event was detected. The genetic map for 98 IMF2s was then deduced based on the crossing information of RILs.

### 5.5 Alignment of sRNA and sRNA cluster to the reference genome

sRNAs were aligned to the Minghui 63 genome and the Minghui 63 genome with SNP sites replaced by Zhenshan 97 sequences using Bowtie (parameters “-v 0 –m 1”) to determine their genomic locations utilizing the same principles used in our previous study [17]. sRNA clusters were aligned to the Minghui 63 reference genome using BLASTN. Alignments with identity ≥ 90% and query coverage ≥ 85% were used to determine the genomic locations of sRNA clusters. Results with more than one alignment hit were discarded.

### 5.6 Mites in the genome of Minghui 63

The sequences of MITEs in the Nipponbare genome were downloaded from the P-MITE database, which were then aligned to the genome of Minghui 63 using BLASTN (Query coverage ≥ 95%, e-value ≤ 1e-5) to annotate the MITEs in the genome of Minghui 63 [17], [43].

### 5.7 Quantification of the expression levels of long inverted repeats in the IMF2 population using mRNA sequencing data

For Bin1556, the sequence of the long inverted repeat (LIR) represented by chr12:22977690–22980803 of the Minghui 63 genome was extracted as the reference genome. Then the mRNA sequencing data of each IMF2 was aligned to the LIR reference by HISAT2 (parameter “-k 1”) allowing one alignment for each sequencing read. The number of aligned reads were then divided by the total sequencing reads of each IMF2 (in millions), which were defined as the expression level of the LIR. For Bin795, the sequences of the LIRs were extracted from the Zhenshan 97 and Minghui 63 genomes. The mRNA sequencing data of IMF2s of Zhenshan 97 genotype were aligned to the LIR from the Zhenshan 97 genome, while the mRNA sequencing data of IMF2s of Minghui 63 genotype were aligned to the LIR from the Minghui 63 genome. The mRNA sequencing data of heterozygote genotype were aligned to the combined LIRs of both genomes. The process of sequence alignment and expression quantification were the same as that for Bin795.

## Declaration of Competing Interest

The authors declare that they have no known competing financial interests or personal relationships that could have appeared to influence the work reported in this paper.

## References

[1] D’Ario M., Griffiths-Jones S., Kim M. (2017). Small RNAs: Big Impact on Plant Development. Trends Plant Sci.

[2] Yu Y., Zhang Y., Chen X., Chen Y. (2019). Plant Noncoding RNAs: Hidden Players in Development and Stress Responses. Annu Rev Cell Dev Biol.

[3] Axtell M.J. (2013). Classification and comparison of small RNAs from plants. Annu Rev Plant Biol.

[4] Sun W., Xu X.H., Li Y., Xie L., He Y. (2020). OsmiR530 acts downstream of OsPIL15 to regulate grain yield in rice. New Phytol.

[5] Zhang J., Zhang H., Srivastava A.K., Pan Y., Bai J. (2018). Knockdown of Rice MicroRNA166 Confers Drought Resistance by Causing Leaf Rolling and Altering Stem Xylem Development. Plant Physiol.

[6] Yang R., Li P., Mei H., Wang D., Sun J. (2019). Fine-Tuning of MiR528 Accumulation Modulates Flowering Time in Rice. Molecular Plant.

[7] Yao S., Yang Z., Yang R., Huang Y., Guo G. (2019). Transcriptional Regulation of miR528 by OsSPL9 Orchestrates Antiviral Response in Rice. Molecular Plant.

[8] Zhang H., Tao Z., Hong H., Chen Z., Wu C. (2016). Transposon-derived small RNA is responsible for modified function of WRKY45 locus. Nat Plants.

[9] Xia R., Chen C., Pokhrel S., Ma W., Huang K. (2019). 24-nt reproductive phasiRNAs are broadly present in angiosperms. Nat Commun.

[10] Fei Q., Yang L., Liang W., Zhang D., Meyers B.C. (2016). Dynamic changes of small RNAs in rice spikelet development reveal specialized reproductive phasiRNA pathways. J Exp Bot.

[11] Bologna N.G., Voinnet O. (2014). The Diversity, Biogenesis, and Activities of Endogenous Silencing Small RNAs in *Arabidopsis*. Annu Rev Plant Biol.

[12] Henderson I.R., Zhang X., Lu C., Johnson L., Meyers B.C. (2006). Dissecting *Arabidopsis thaliana* DICER function in small RNA processing, gene silencing and DNA methylation patterning. Nat Genet.

[13] Wei L., Gu L., Song X., Cui X., Lu Z. (2014). Dicer-like 3 produces transposable element-associated 24-nt siRNAs that control agricultural traits in rice. Proc Natl Acad Sci.

[14] Ye R., Chen Z., Lian B., Rowley M.J., Xia N. (2016). A Dicer-Independent Route for Biogenesis of siRNAs that Direct DNA Methylation in *Arabidopsis*. Mol Cell.

[15] Fang X., Zhao G., Zhang S., Li Y., Gu H. (2019). Chloroplast-to-Nucleus Signaling Regulates MicroRNA Biogenesis in *Arabidopsis*. Dev Cell.

[16] Wang J., Yao W., Zhu D., Xie W., Zhang Q. (2015). Genetic basis of sRNA quantitative variation analyzed using an experimental population derived from an elite rice hybrid. Elife.

[17] Zhang J., Chen L.-L., Xing F., Kudrna D.A., Yao W. (2016). Proceedings of the National Academy of Sciences.

[18] Wang J., Yao W., Zhu D., Xie W., Zhang Q. (2015). Data from: Genetic basis of sRNA quantitative variation analyzed using an experimental population derived from an elite rice hybrid. Dryad Data Repository..

[19] Haley C.S., Knott S.A. (1992). A simple regression method for mapping quantitative trait loci in line crosses using flanking markers. Heredity (Edinb).

[20] Broman K.W., Speed T.P. (2002). A model selection approach for the identification of quantitative trait loci in experimental crosses. Journal of the Royal Statistical Society: Series B (Statistical Methodology).

[21] Manichaikul A., Moon J.Y., Sen S., Yandell B.S., Broman K.W. (2009). A model selection approach for the identification of quantitative trait loci in experimental crosses, allowing epistasis. Genetics.

[22] Altschul S.F., Gish W., Miller W., Myers E.W., Lipman D.J. (1990). Basic local alignment search tool. J Mol Biol.

[23] Langmead B., Trapnell C., Pop M., Salzberg S.L. (2009). Ultrafast and memory-efficient alignment of short DNA sequences to the human genome. Genome Biol.

[24] Anders S., Huber W. (2010). Differential expression analysis for sequence count data. Genome Biol.

[25] Arikit S., Zhai J., Meyers B.C. (2013). Biogenesis and function of rice small RNAs from non-coding RNA precursors. Curr Opin Plant Biol.

[26] Gruber A.R., Lorenz R., Bernhart S.H., Neuböck R., Hofacker I.L. (2008). The Vienna RNA websuite. Nucleic Acids Res.

[27] Wainberg M., Sinnott-Armstrong N., Mancuso N., Barbeira A.N., Knowles D.A. (2019). Opportunities and challenges for transcriptome-wide association studies. Nat Genet.

[28] Mi S., Cai T., Hu Y., Chen Y., Hodges E. (2008). Sorting of small RNAs into *Arabidopsis* argonaute complexes is directed by the 5' terminal nucleotide. Cell.

[29] Takeda A., Iwasaki S., Watanabe T., Utsumi M., Watanabe Y. (2008). The Mechanism Selecting the Guide Strand from Small RNA Duplexes is Different Among Argonaute Proteins. Plant Cell Physiol.

[30] Azevedo J., Garcia D., Pontier D., Ohnesorge S., Yu A. (2010). Argonaute quenching and global changes in Dicer homeostasis caused by a pathogen-encoded GW repeat protein. Genes Dev.

[31] Mallory A.C., Vaucheret H. (2009). ARGONAUTE 1 homeostasis invokes the coordinate action of the microRNA and siRNA pathways. EMBO Rep.

[32] Kreuze J.F., Perez A., Untiveros M., Quispe D., Fuentes S. (2009). Complete viral genome sequence and discovery of novel viruses by deep sequencing of small RNAs: a generic method for diagnosis, discovery and sequencing of viruses. Virology.

[33] Warren R.L., Sutton G.G., Jones S.J., Holt R.A. (2007). Assembling millions of short DNA sequences using SSAKE. Bioinformatics.

[34] Jeck W.R., Reinhardt J.A., Baltrus D.A., Hickenbotham M.T., Magrini V. (2007). Extending assembly of short DNA sequences to handle error. Bioinformatics.

[35] Zerbino D.R., Birney E. (2008). Velvet: Algorithms for de novo short read assembly using de Bruijn graphs. Genome Res.

[36] Hernandez D., François P., Farinelli L., Østerås M., Schrenzel J. (2008). De novo bacterial genome sequencing: Millions of very short reads assembled on a desktop computer. Genome Res.

[37] Dohm J.C., Lottaz C., Borodina T., Himmelbauer H. (2007). SHARCGS, a fast and highly accurate short-read assembly algorithm for de novo genomic sequencing. Genome Res.

[38] Pertea G., Huang X., Liang F., Antonescu V., Sultana R. (2003). TIGR Gene Indices clustering tools (TGICL): a software system for fast clustering of large EST datasets. Bioinformatics.

[39] Yao W., Li G., Zhao H., Wang G., Lian X. (2015). Exploring the rice dispensable genome using a metagenome-like assembly strategy. Genome Biol.

[40] Zhou G., Chen Y., Yao W., Zhang C., Xie W. (2012). Genetic composition of yield heterosis in an elite rice hybrid. Proc Natl Acad Sci.

[41] Xie W., Feng Q., Yu H., Huang X., Zhao Q. (2010). Parent-independent genotyping for constructing an ultrahigh-density linkage map based on population sequencing. Proc Natl Acad Sci.

[42] Yu H., Xie W., Wang J., Xing Y., Xu C. (2011). Gains in QTL detection using an ultra-high density SNP map based on population sequencing relative to traditional RFLP/SSR markers. PLoS ONE.

[43] Chen J., Hu Q., Zhang Y., Lu C., Kuang H. (2014). P-MITE: a database for plant miniature inverted-repeat transposable elements. Nucleic Acids Res.

[44] Yu Y., Ouyang Y., Yao W. (2018). shinyCircos: an R/Shiny application for interactive creation of Circos plot. Bioinformatics.

