# Supplementary material for: Features of sRNA biogenesis in rice revealed by genetic dissection of sRNA expression level
Source: Comput Struct Biotechnol J. 2020 Oct 23;18:3207–16. doi: 10.1016/j.csbj.2020.10.012 (PMC7649420; doi:10.1016/j.csbj.2020.10.012)
Supplement: Supplementary data 1 [file mmc1.docx]

**Supplementary figures**


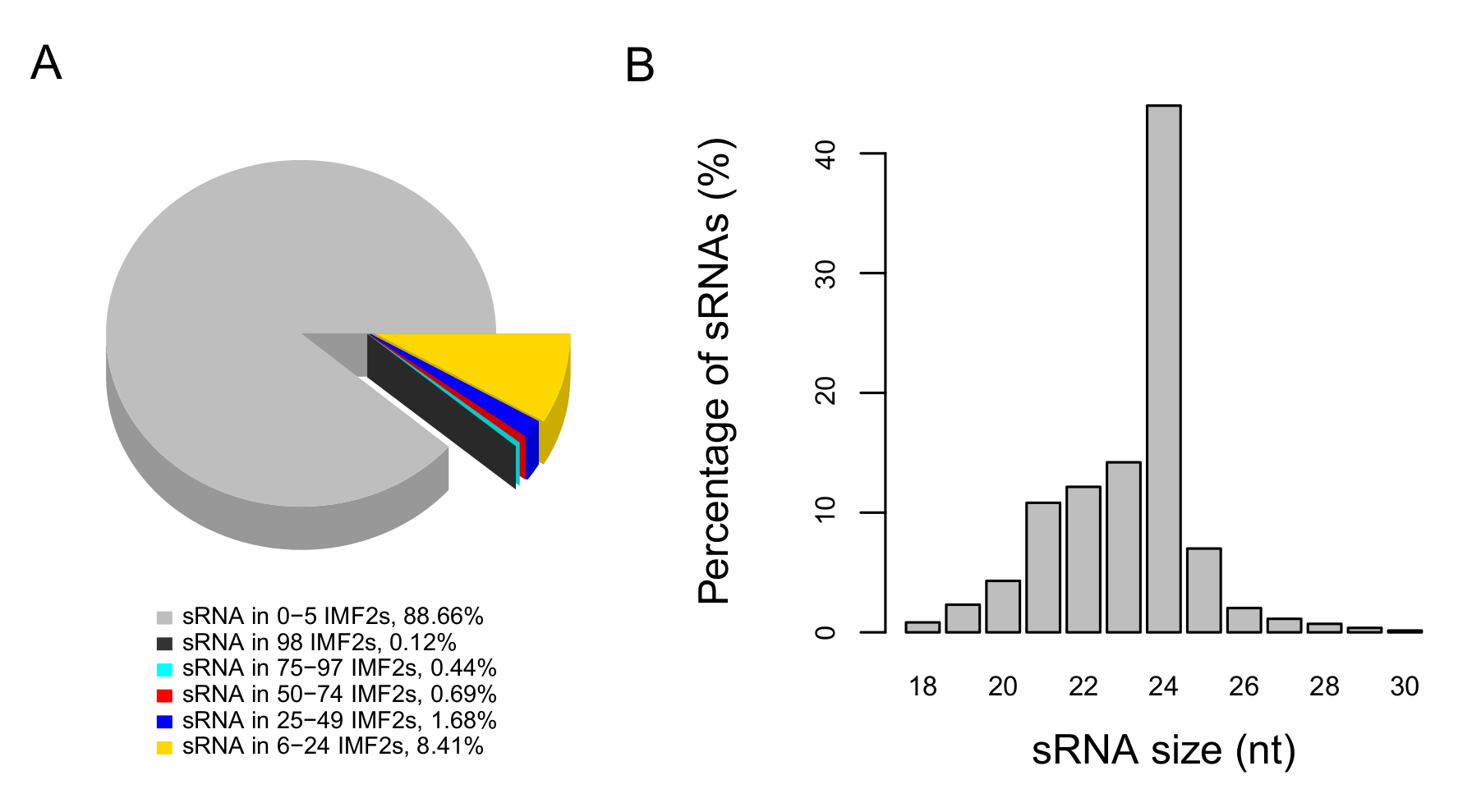


**Figure S1. The distribution of sRNAs across the IMF2 population and the size of sRNAs.**

(A) The distribution of sRNAs across the IMF2 population. (B) The percentages of sRNAs of different sizes.


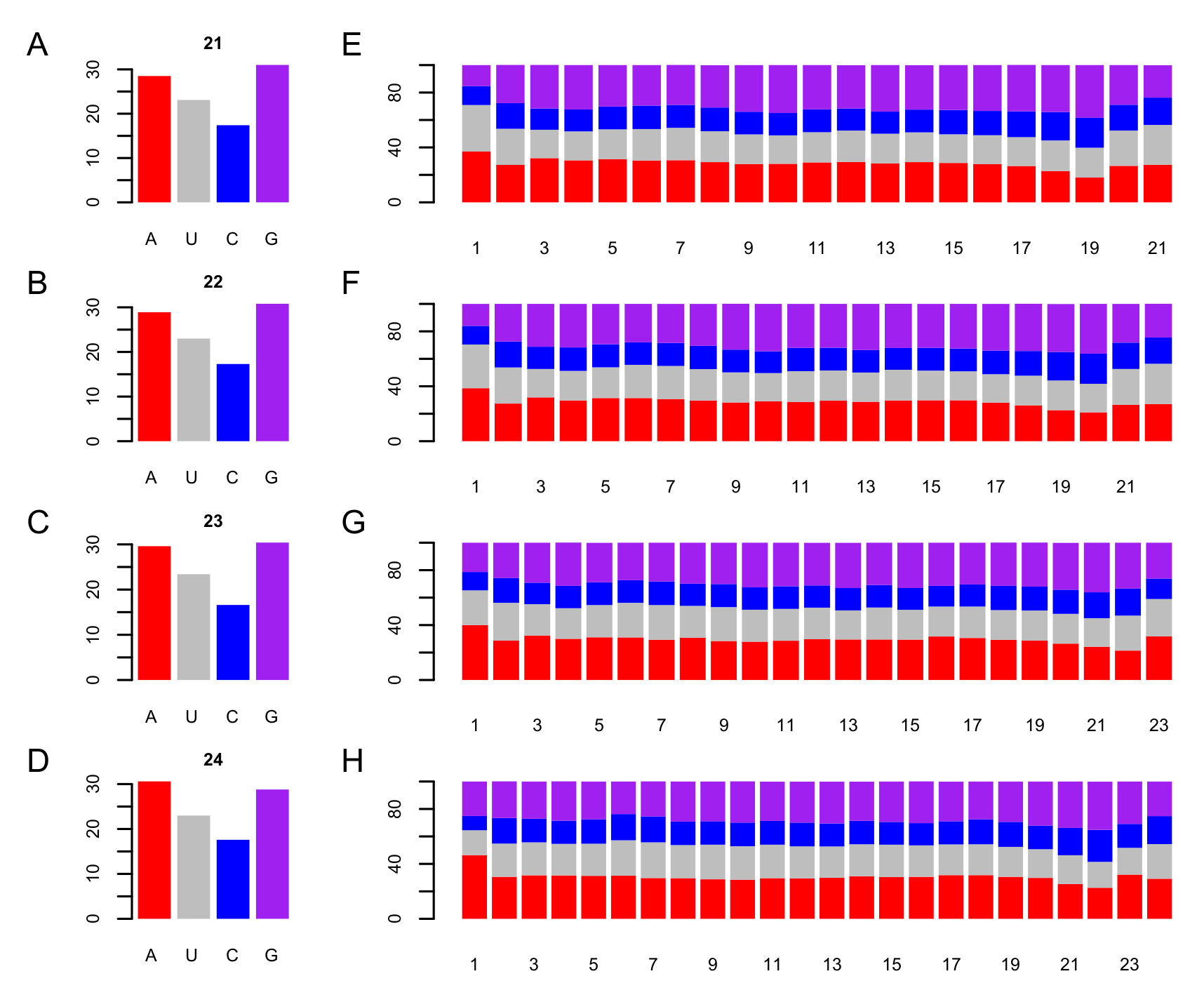


**Figure S2. The nucleotide composition of sRNAs of different sizes.**

The percentage of different nucleotides at all base positions of 21-nt (A), 22-nt (B), 23-nt (C) and 24-nt (D) sRNAs. The percentage of different nucleotides at each base position of 21-nt (E), 22-nt (F), 23-nt (G) and 24-nt (H) sRNAs. Each vertical bar represents a base position. The length of different sections of each bar indicate the percentage of different nucleotides. Red, A; grey, U; blue, C; purple, G.


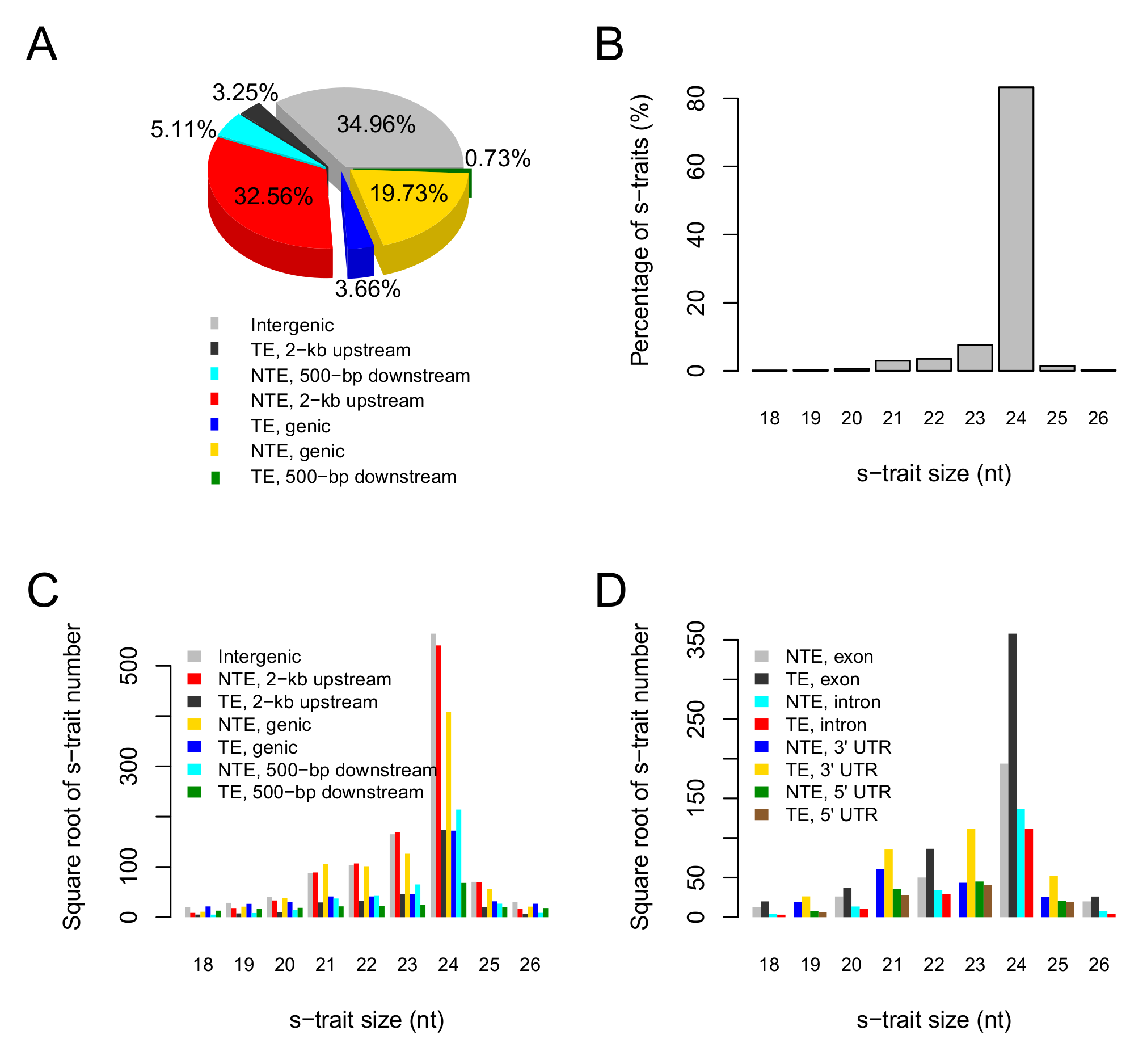


**Figure S3. The distribution of s-traits in different genomic regions across the IMF2 population.**

(A) The distribution of s-traits aligned to the genic region, 2-kb upstream, and 500-bp downstream of annotated genes, as well as the intergenic regions. TE: transposons; NTE: non-transposon genes. (B) The percentages of s-traits of different sizes. (C) The distribution of s-traits of different sizes in different genomic regions. (D) The distribution of s-traits of different sizes in different portions of genic regions.


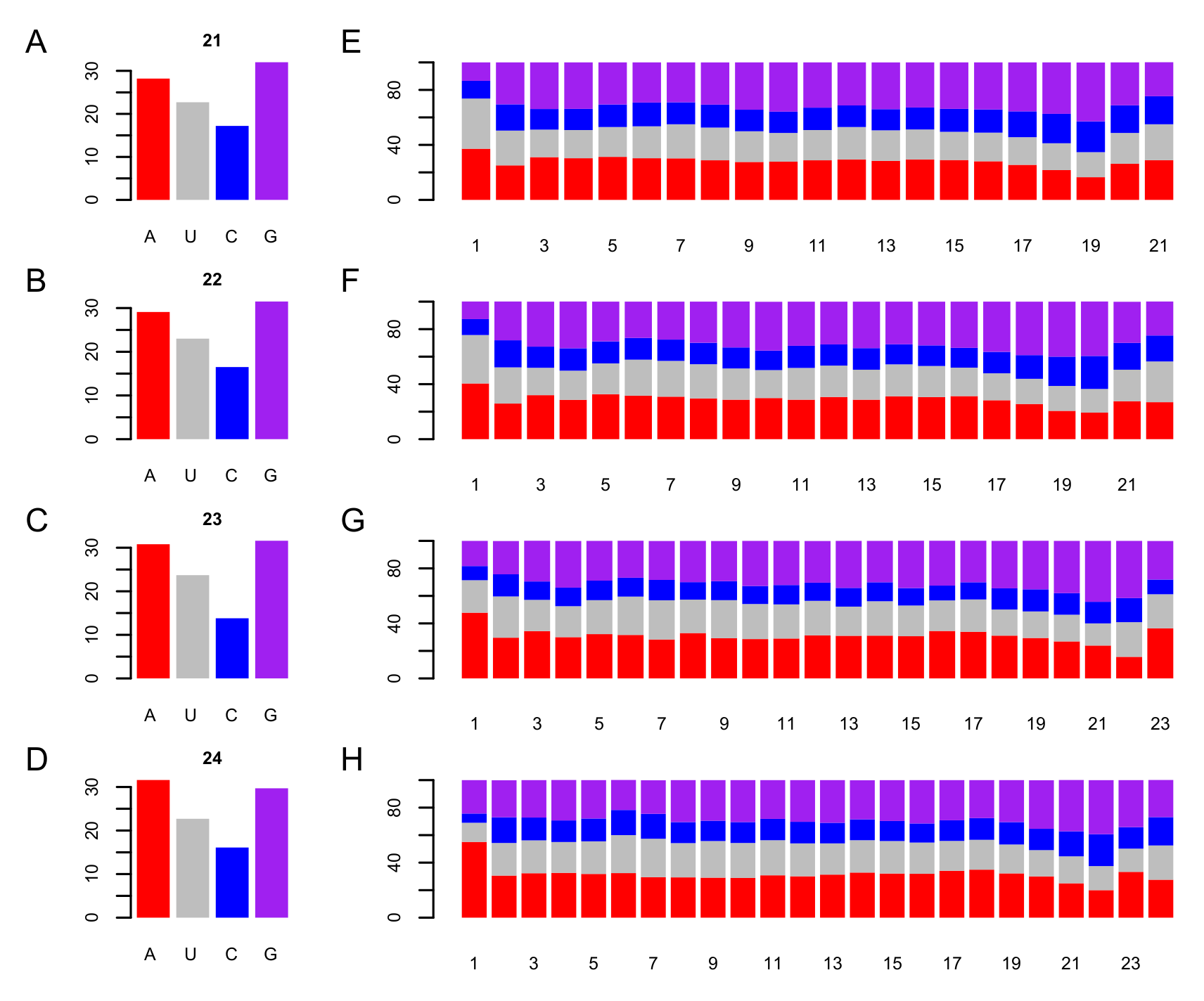


**Figure S4. The nucleotide composition of s-traits of different sizes.**

The percentage of different nucleotides at all base positions of 21-nt (A), 22-nt (B), 23-nt (C) and 24-nt (D) s-traits. The percentage of different nucleotides at each base position of 21-nt (E), 22-nt (F), 23-nt (G) and 24-nt (H) s-traits. Each vertical bar represents a base position. The length of different sections of each bar indicate the percentage of different nucleotides. Red, A; grey, U; blue, C; purple, G.


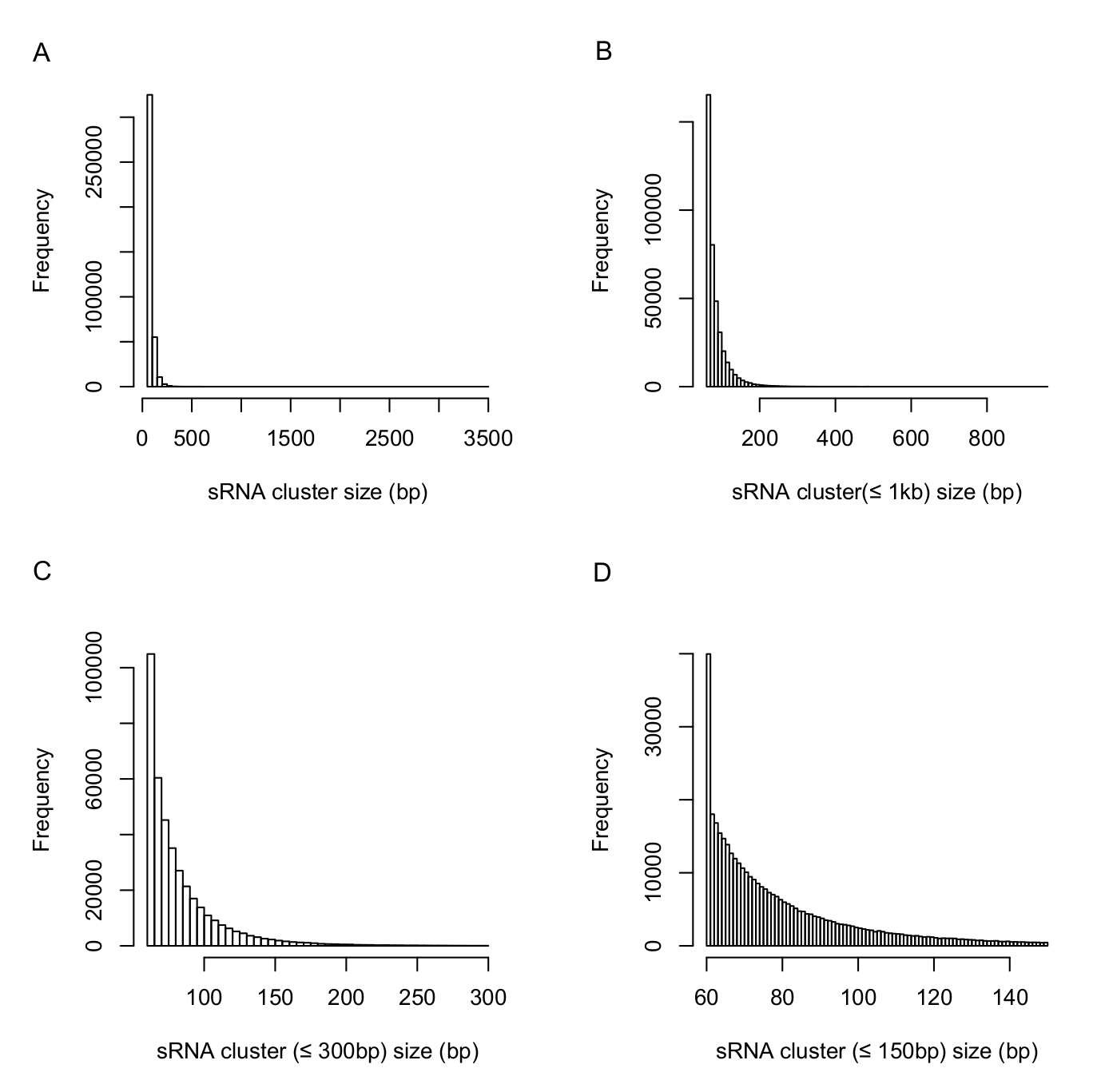


**Figure S5. Distribution of sRNA cluster sizes.**

(A) Distribution of the sizes of all 394,965 sRNA clusters. (B) Distribution of the sizes of sRNA clusters not longer than 1 kb. (C) Distribution of the sizes of sRNA clusters not longer than 300 bp. (D) Distribution of the sizes of sRNA clusters not longer than 150 bp.

**
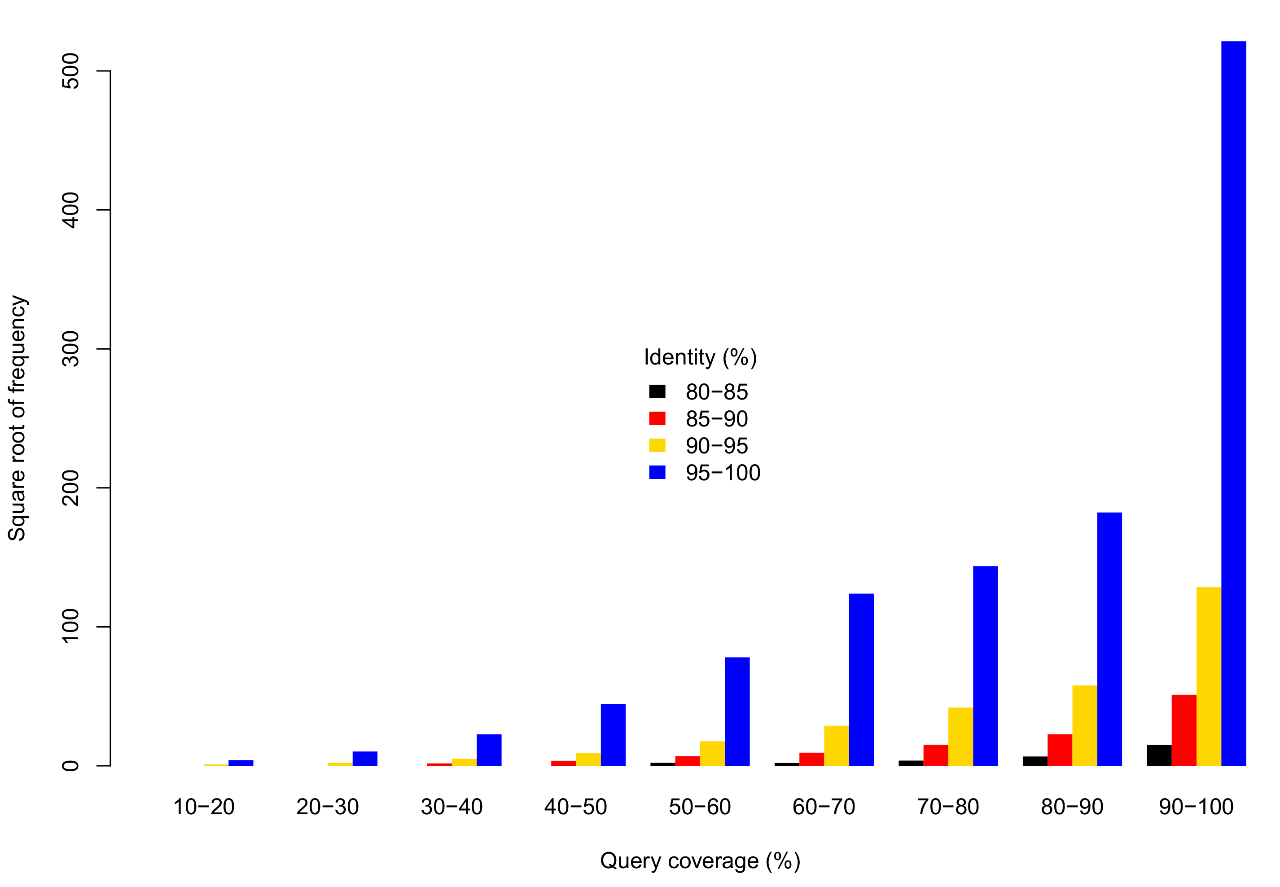
**

**Figure S6. Evaluation of the assembly of sRNA reads based on the alignment of the assembly to the Minghui 63 genome using BLASTN.**

Query coverage for a contig is defined as the matched length between the contig and the Minghui 63 genome divided by the length of the contig. The contigs are categorized as 10 groups based on the query coverage of the alignment and contigs in each group are further labeled with different colors based on the identity of the alignment. 0-10, from 0 (excluded) to 10 (included).


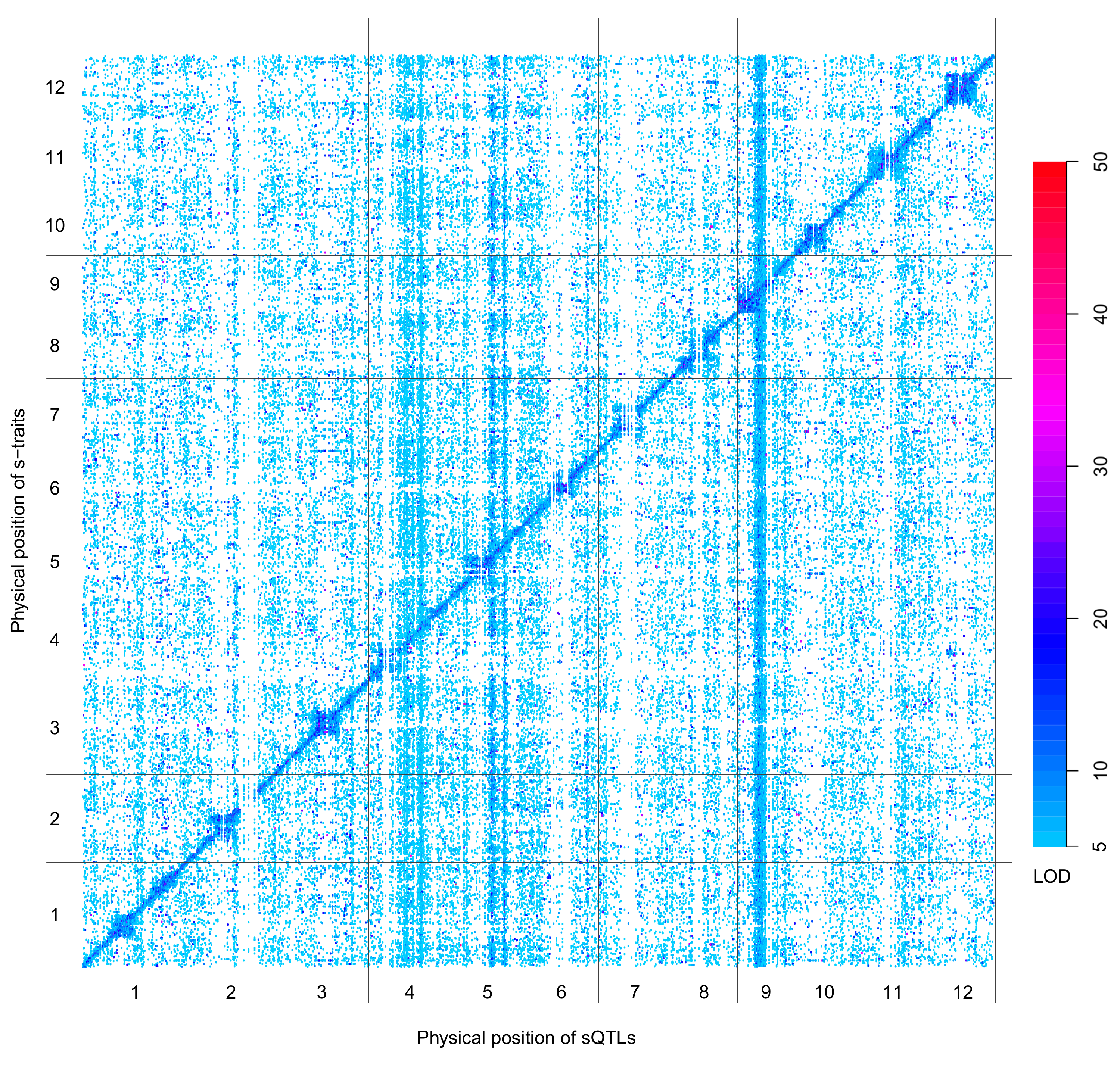


**Figure S7. sQTLs for the 306,371 s-traits.**

The color key shows the LOD value. X-axis, the physical position of sQTLs along the 12 chromosomes. Y-axis, the physical position of s-traits.


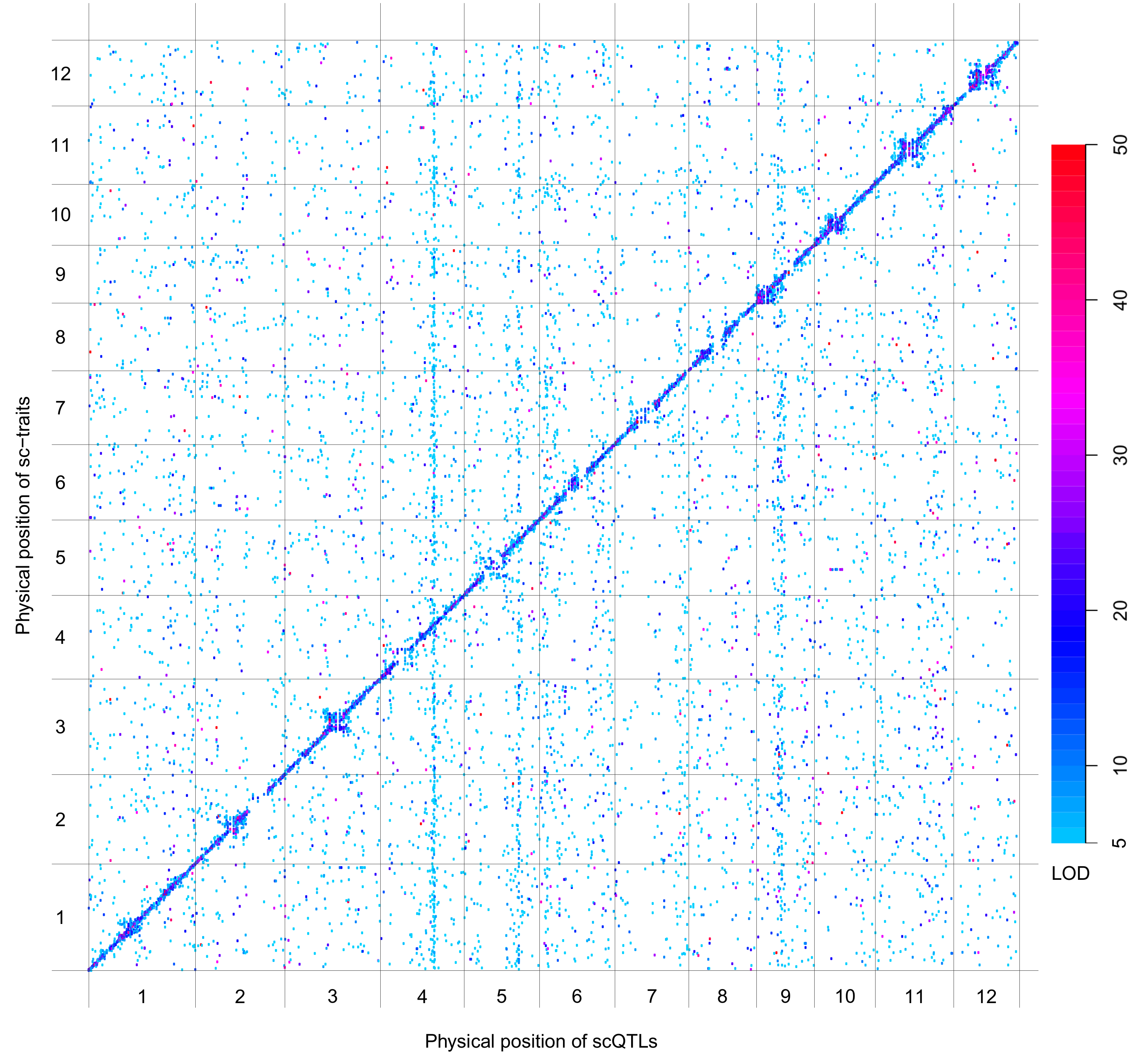


**Figure S8. scQTLs for the 22,070 sc-traits.**

The color key shows the LOD value. X-axis, the physical position of scQTLs along the 12 chromosomes. Y-axis, the physical position of sc-traits.


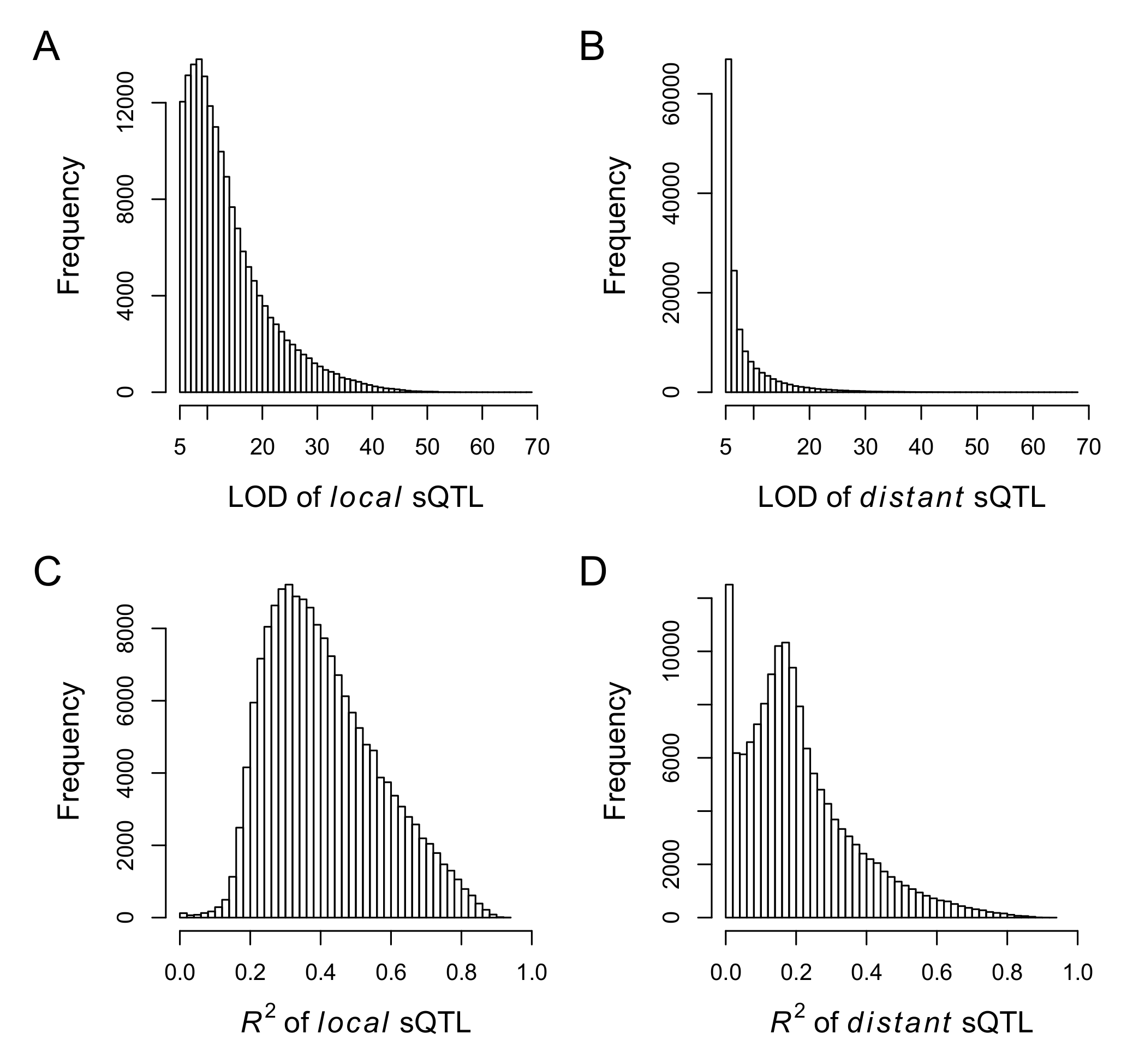


**Figure S9. The LOD values of sQTLs and the expression variations explained by sQTLs.**

(A) The LOD values for *local*-sQTLs. (B) The LOD values for *distant*-sQTLs. (C) Expression variation explained by *local*-sQTL. (D) Expression variation explained by *distant*-sQTL.


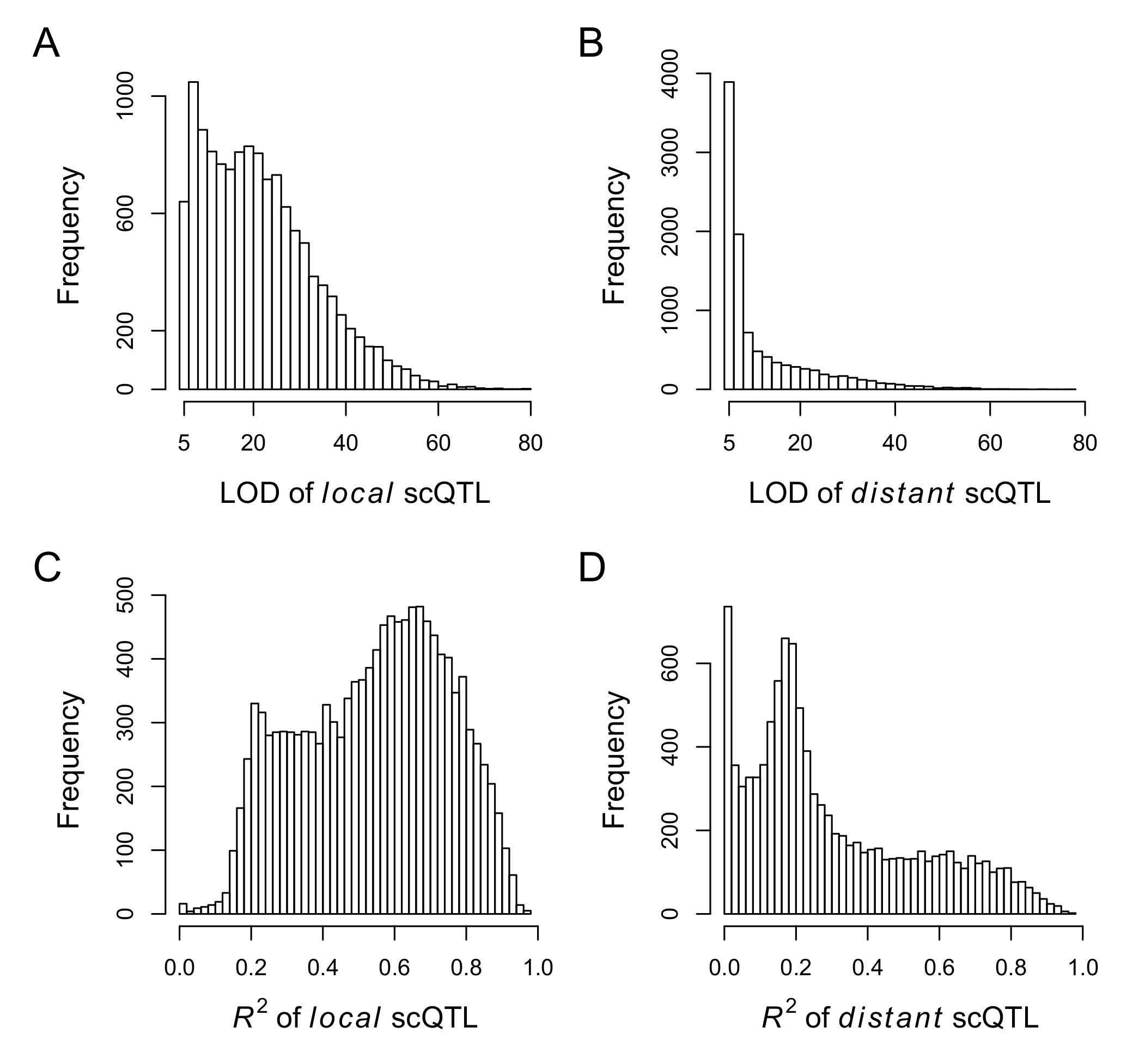


**Figure S10. The LOD values of scQTLs and the expression variations explained by scQTLs.**

(A) The LOD values for *local*-scQTLs. (B) The LOD values for *distant*-scQTLs. (C) Expression variation explained by *local*-scQTL. (D) Expression variation explained by *distant*-scQTL.


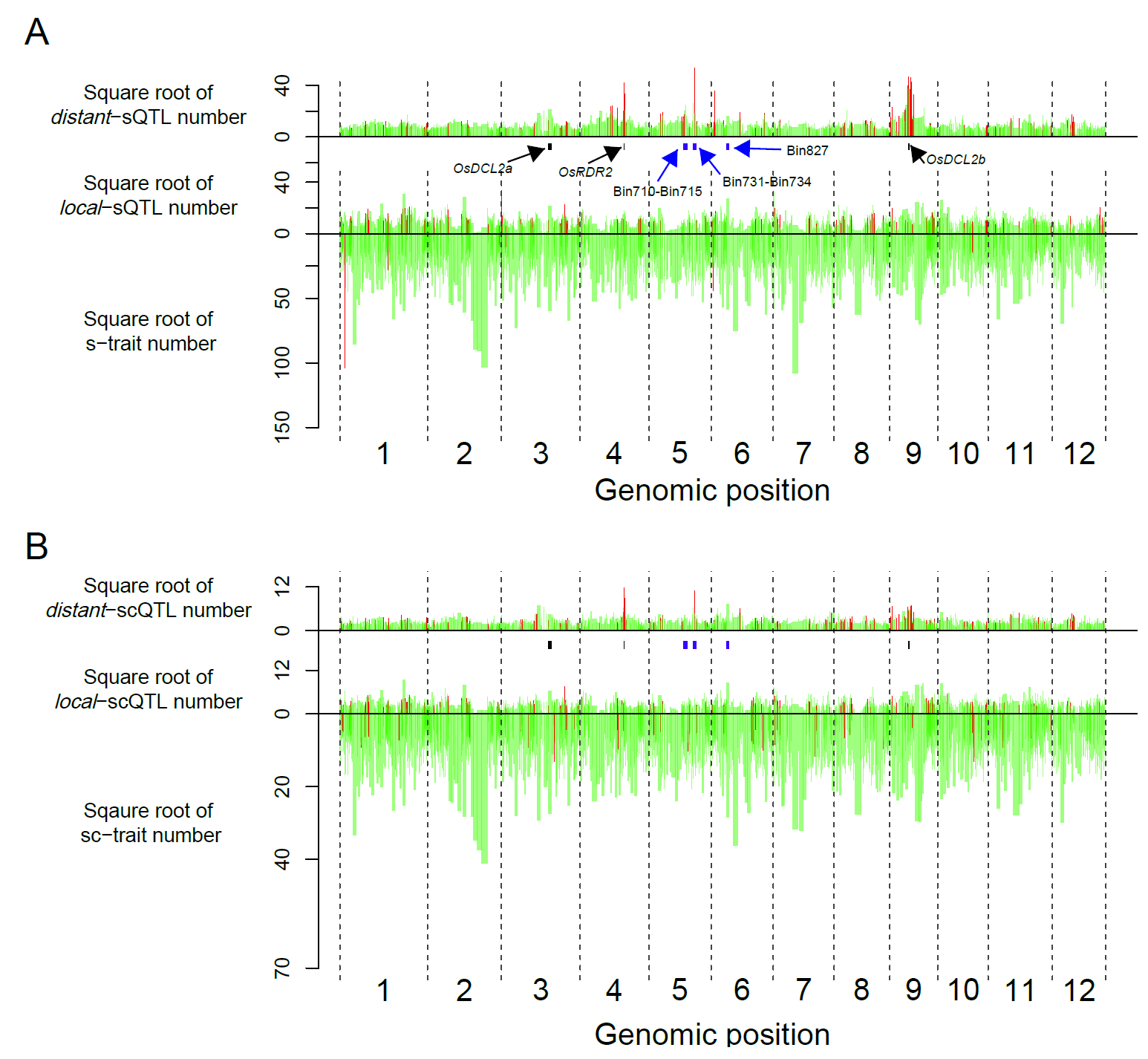


**Figure S11. Hotspots of traits and QTLs.**

(A) s-traits and sQTLs. (B) sc-traits and scQTLs.

The 1,567 bins are placed from left to right according to their genomic locations. The width of each bar represents the size of the bin. The chromosome identifiers are labeled below each plot. Different chromosomes are separated by grey dashed lines. Bins in red color are trait or QTL hotspots. Bins harboring sRNA biogenesis genes are marked with black rectangles.


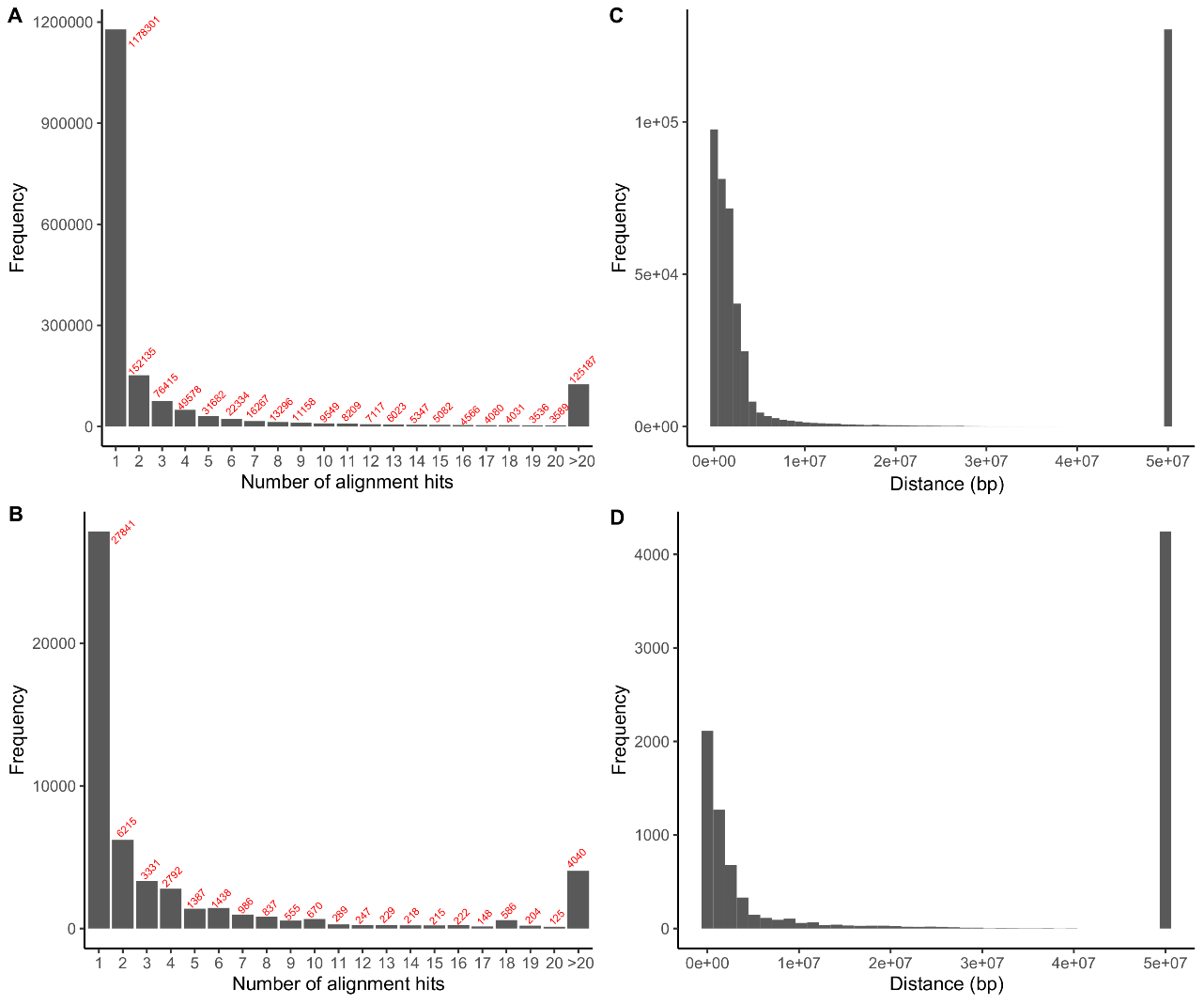


**Figure S12. Alignment of s-traits to the parental genomes and comparisons of the alignments with the genomic positions of sQTLs.**

(A) 1,737,482 s-traits aligned without mismatch to one or multiple positions of the parental genomes. Each bar represents a group of s-traits aligned to specific number of positions of the parental genomes. (B) 52,575 s-traits aligned with one mismatch to one or multiple positions of the parental genomes. (C) The minimum distance between the aligned positions of a s-trait (of all 1,737,482 s-traits in figure A) and the sQTL regulating its expression. The distance of 50 Mb indicates that the sRNA and the corresponding sQTL are located on two different chromosomes. (D) The minimum distance between the aligned positions of a s-trait (of all 52,575 s-traits in figure B) and the sQTL regulating its expression.


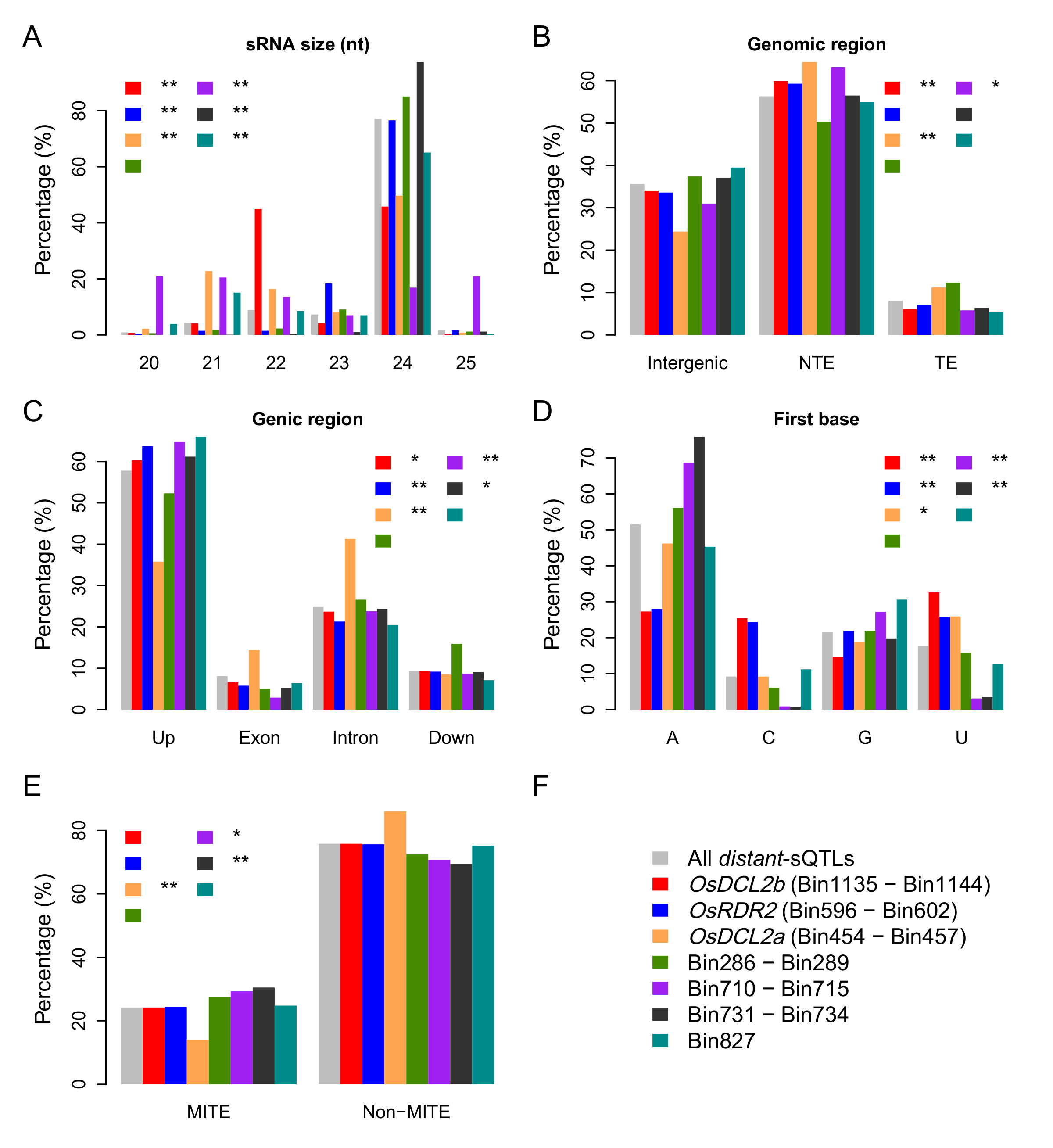


**Figure S13. Diverse features of uniquely mapped sRNAs regulated by different *distant*-sQTL hotspots.**

(A) The percentage of sRNAs of varying size regulated by different *distant*-sQTL hotspots. (B) The distribution of sRNAs from the intergenic region, the TE and NTE regions regulated different *distant*-sQTL hotspots. (C) The genic distribution of sRNAs regulated by different *distant*-sQTL hotspots. (D) The preference of the first base at the 5' end for sRNAs regulated by different *distant*-sQTL hotspots. (E) The percentage of sRNAs from MITEs and non-MITEs regulated by different *distant*-sQTL hotspots. Different *distant*-sQTL hotspots are represented with different colors as indicated in (F). The percentage of sRNAs with different features regulated by *distant*-sQTL hotspots are compared with that of all sRNAs with *distant*-sQTLs using chi-squared test. *, *p*-value <1e-5. **, *p*-value <1e-10.


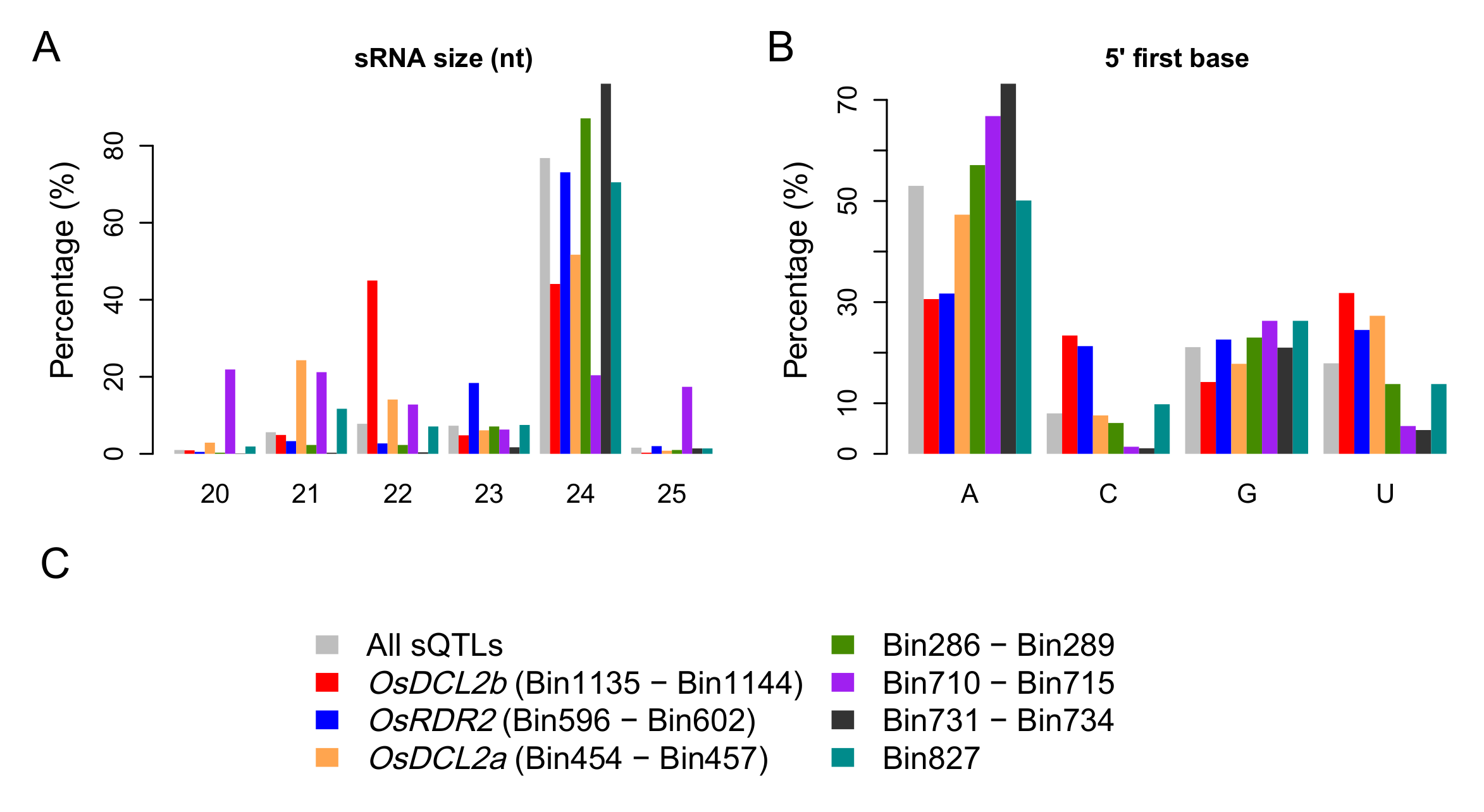


**Figure S14. Diverse features of all sRNAs regulated by different sQTL hotspots.**

(A) The percentage of sRNAs of varying size regulated by different sQTL hotspots. (B) The preference of the first base at the 5' end for sRNAs regulated by different sQTL hotspots. Different sQTL hotspots are represented with different colors as indicated in (C).


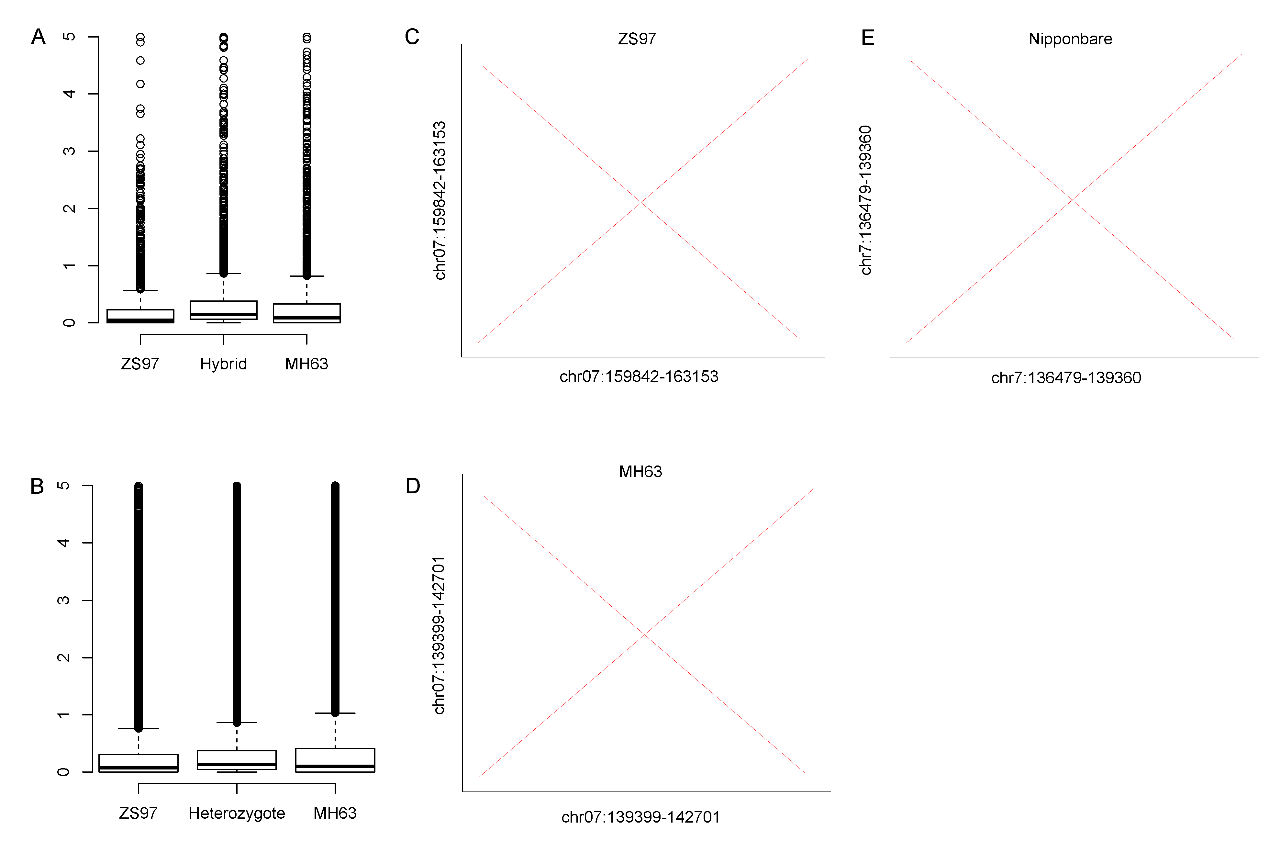


**Figure S15. Expressions of sRNAs regulated by Bin903 and structures of the candidate precursors of sRNAs regulated by Bin903 in the parental genomes.**

(A) Expression values of sRNAs regulated by Bin903 in Zhenshan 97 (ZS97), Minghui 63 (MH63) and the hybrid. (B) Expression values of sRNAs regulated by Bin903 in IMF2s with different genotypes. ZS97, the Zhenshan 97 genotype. MH63, the Minghui 63 genotype. Heterozygote, heterozygote genotype. (C) Self-alignment of chr07:159842-163153 of the Zhenshan 97 genome. (D) Self-alignment of chr07:139399-142701 of the Minghui 63 genome. (E) Self-alignment of chr07:136479-139360 of the Nipponbare genome. The majority of sRNAs regulated by Bin903 could be aligned to the genomic regions of Zhenshan 97, Minghui 63 and Nipponbare shown in (C), (D) and (E), respectively.


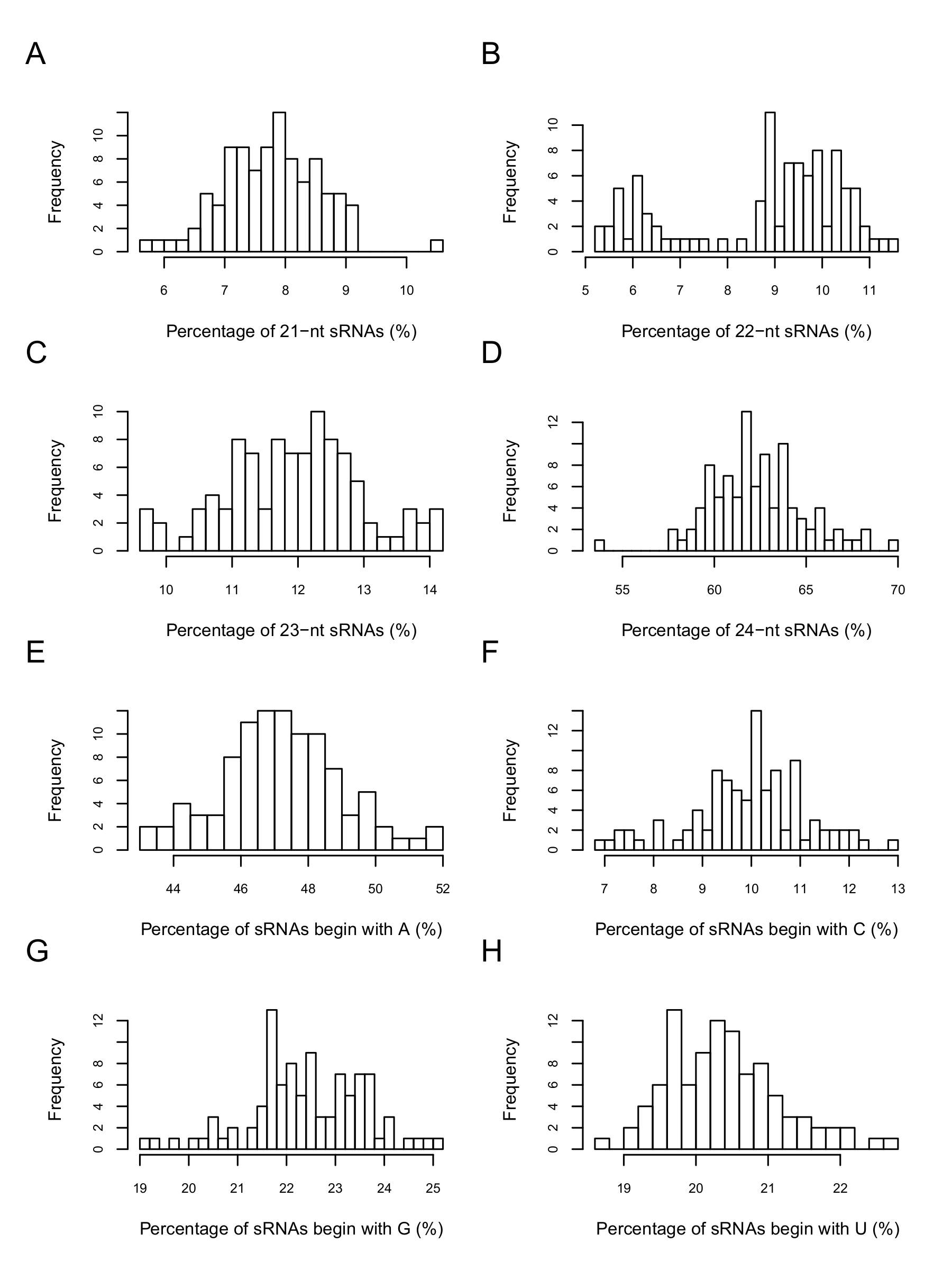


**Figure S16. The distribution of the percentage of varying species of sRNAs in all unique sRNA reads for different IMF2s.**


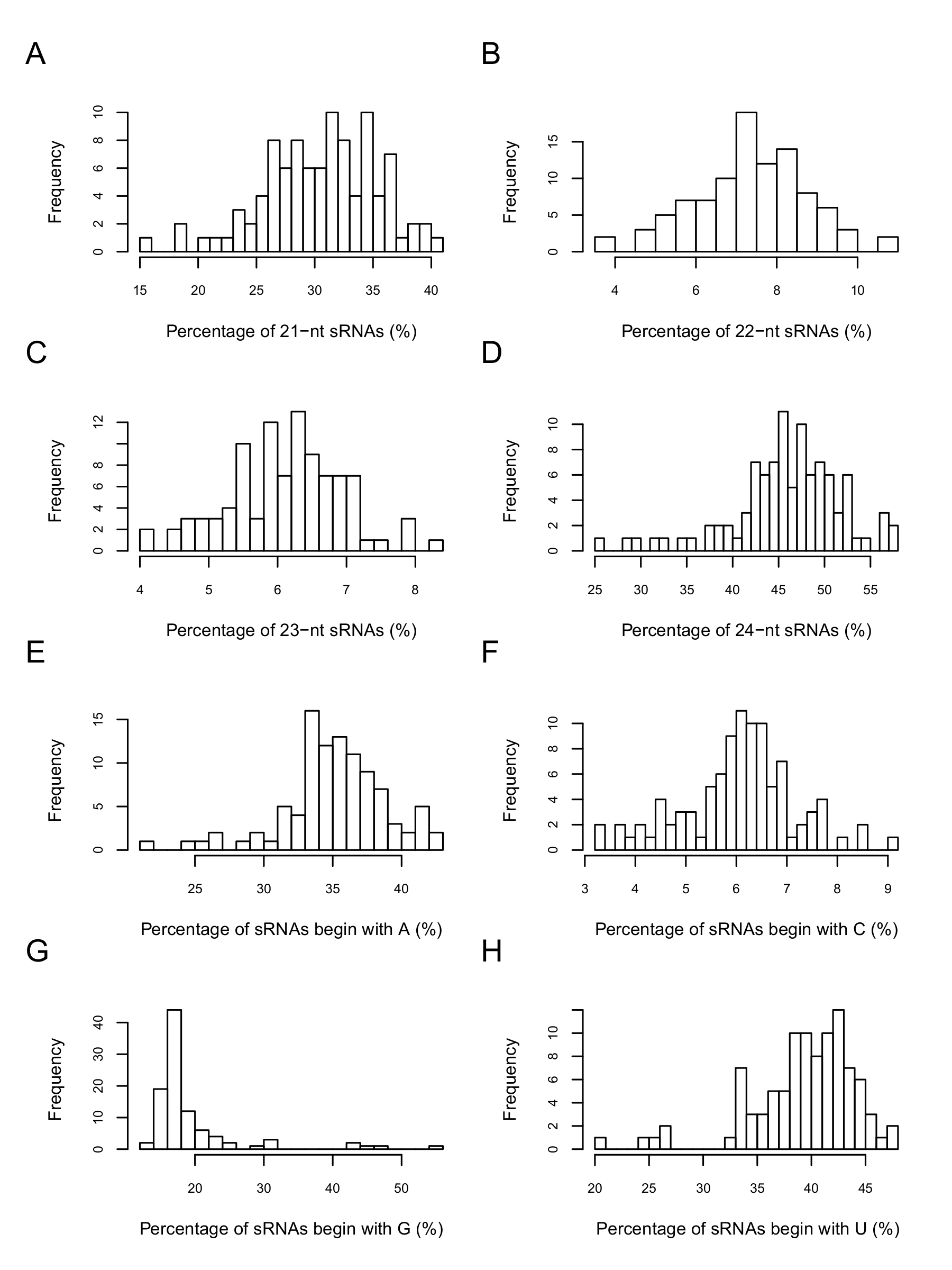


**Figure S17. The distribution of the percentage of varying species of sRNAs in all redundant sRNA reads for different IMF2s.**
